# Fusion protein of retinol-binding protein and albumin domain III reduces liver fibrosis

**DOI:** 10.15252/emmm.201404527

**Published:** 2015-04-11

**Authors:** Hongsik Lee, Hyeyeun Jeong, Sangeun Park, Wonbaek Yoo, Soyoung Choi, Kyungmin Choi, Min-Goo Lee, Mihwa Lee, DaeRyong Cha, Young-Sik Kim, Jeeyoung Han, Wonkon Kim, Sun-Hwa Park, Junseo Oh

**Affiliations:** 1 Department of Internal Medicine, College of Medicine, Korea UniversitySeoul, Korea; 2 Department of Anatomy, College of Medicine, Korea UniversitySeoul, Korea; 3 Department of Physiology, College of Medicine, Korea UniversitySeoul, Korea; 4 Department of Nephrology, College of Medicine, Korea UniversitySeoul, Korea; 5 Department of Pathology, College of Medicine, Korea UniversitySeoul, Korea; 6 Department of Pathology, Inha University HospitalIncheon, Korea; 7 Medical Proteomics Research Center, KRIBBDaejeon, Korea

**Keywords:** albumin, anti-fibrotic drug, fibrosis, hepatic stellate cell, retinoic acid

## Abstract

Activated hepatic stellate cells (HSCs) play a key role in liver fibrosis, and inactivating HSCs has been considered a promising therapeutic approach. We previously showed that albumin and its derivative designed for stellate cell-targeting, retinol-binding protein–albumin domain III fusion protein (referred to as R-III), inactivate cultured HSCs. Here, we investigated the mechanism of action of albumin/R-III in HSCs and examined the anti-fibrotic potential of R-III *in vivo*. R-III treatment and albumin expression downregulated retinoic acid (RA) signaling which was involved in HSC activation. RA receptor agonist and retinaldehyde dehydrogenase overexpression abolished the anti-fibrotic effect of R-III and albumin, respectively. R-III uptake into cultured HSCs was significantly decreased by siRNA-STRA6, and injected R-III was localized predominantly in HSCs in liver. Importantly, R-III administration reduced CCl_4_- and bile duct ligation-induced liver fibrosis. R-III also exhibited a preventive effect against CCl_4_-inducd liver fibrosis. These findings suggest that the anti-fibrotic effect of albumin/R-III is, at least in part, mediated by downregulation of RA signaling and that R-III is a good candidate as a novel anti-fibrotic drug.

## Introduction

Liver fibrosis, characterized by excessive production and deposition of extracellular matrix (ECM) components, is a common response to chronic liver injury (Hernandez-Gea & Friedman, 2011). There is overwhelming evidence that activated hepatic stellate cells (HSCs) are major producers of fibrotic neomatrix, although additional cellular sources, such as portal myofibroblasts, bone marrow-derived cells, and epithelial-to-mesenchymal transition (EMT), have been reported (Forbes & Parola, 2011). In normal liver, HSCs remain quiescent and store approximately 70% of the body's retinoid as retinyl ester in cytoplasmic lipid droplets. However, upon liver injury, quiescent HSCs become activated and transform into myofibroblast-like cells, which is invariably associated with loss of cytoplasmic vitamin A-containing lipid droplets, positive staining for α-smooth muscle actin (α-SMA), and greatly increased synthesis of ECM proteins (Friedman, 2008). Thus, HSCs are considered an attractive target for anti-fibrotic therapies (Li *et al*, 2008). Despite extensive investigations, there is, however, no effective therapy for liver fibrosis and end-stage cirrhosis, except for the removal of the causative agent and organ transplantation.

Retinoids (vitamin A and its metabolites) regulate multiple physiological activities, such as vision, morphogenesis, cell proliferation, and differentiation (Sporn *et al*, 1994). Vitamin A (retinol), acquired from the diet, is transported to the liver and taken up by hepatocytes as a chylomicron remnant. It has been suggested that retinol-binding protein (RBP) plays a role in the transfer of retinol from hepatocytes to HSCs via a RBP receptor STRA6 (Kawaguchi *et al*, 2007; Senoo *et al*, 2010). Upon HSC activation, some retinoid contents are oxidized to retinaldehyde and to retinoic acid (RA), a finding that is supported by the fact that all-*trans* retinoic acid level is increased in activated stellate cells compared with pre-activated stellate cells, whereas the contents of retinyl ester and retinol decrease (Okuno *et al*, 1999; Radaeva *et al*, 2007; D'Ambrosio *et al*, 2011). The diverse effects of retinoids are primarily mediated by two families of nuclear receptors, retinoic acid receptor (RAR) and retinoid X receptor (RXR) (Huang *et al*, 2014).

Albumin is an abundant multifunctional plasma protein synthesized primarily by liver cells (Evans, 2002). It is comprised of three homologous domains, each formed by two smaller subdomains (Curry *et al*, 1998). Crystallographic analysis has revealed that albumin has five high-affinity fatty acid binding sites, three of which are asymmetrically distributed in domain III (Curry *et al*, 1998; Simard *et al*, 2005). In a previous study, we showed that albumin is endogenously expressed in quiescent HSCs and that albumin expression inhibits HSC activation, which requires its intact fatty acid binding sites (Kim *et al*, 2009; Park *et al*, 2012). For therapeutic purposes, we constructed a recombinant fusion protein R-III, in which albumin domain III, retaining the anti-fibrotic activity, was fused to the C-terminus of RBP4. RBP4 was adopted for stellate cell-targeting delivery (Gjoen *et al*, 1987; Senoo *et al*, 1990). HPLC-purified R-III protein successfully entered and inactivated HSCs *in vitro* (Park *et al*, 2012). In this study, we demonstrated that R-III administration alleviated CCl_4_- and bile duct ligation-induced liver fibrosis and that this anti-fibrotic effect was associated with HSC inactivation, at least in part, mediated by downregulation of RA signaling.

## Results

### Retinoic acid signaling is involved in HSC activation

**Figure 1:**
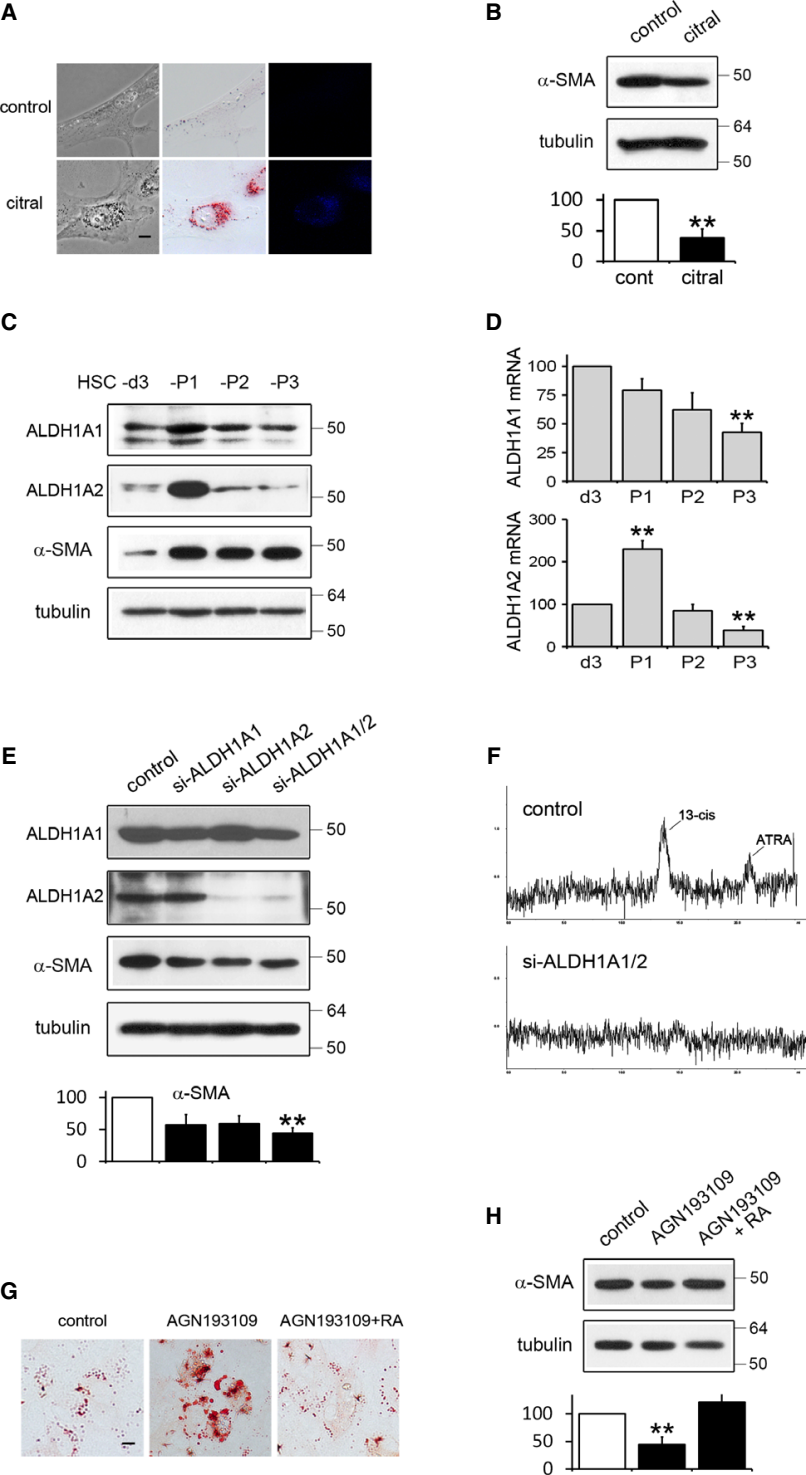
Retinoic acid signaling is involved in HSC activation

The effects of exogenous retinoic acid (RA) on HSCs or liver fibrosis are controversial; several studies showed that RA inactivated HSCs and alleviated hepatic fibrosis (Wang *et al*, 2007; He *et al*, 2011), while other reports showed the opposite (Okuno *et al*, 1997, 2002). There is no clear answer for these conflicting results at the moment. Thus, we sought to examine the role of endogenous RA in HSC activation. HSCs were isolated from rat liver, and hepatocyte contamination was assessed by the expression of the hepatocyte-specific marker tyrosine aminotransferase (TAT) (Supplementary Fig S1). HSCs after passage 1 (HSCs-P1; activated HSCs) were incubated with citral, an inhibitor of retinaldehyde dehydrogenases (also known as aldehyde dehydrogenases ALDH) that irreversibly convert retinaldehyde to RA, and examined for changes. Citral treatment increased autofluorescent lipid droplets (Fig1A) and decreased α-SMA levels (Fig1B), a marker of HSC activation, which are the two most prominent features of inactivated HSCs. Citral treatment did not appear to be toxic as the cells grew normally after it was removed. We then examined the expression pattern of ALDH1A1 and ALDH1A2, responsible for RA synthesis in HSCs (Radaeva *et al*, 2007), during the culture activation of HSCs. Western blot analysis revealed that ALDH1A1 is expressed both in HSCs at day 3 after plating (HSCs-d3; pre-activated HSCs) and activated HSCs but relatively higher expression is found in HSCs-P1 (Fig1C), as previously reported (Radaeva *et al*, 2007). Real-time PCR analysis, however, showed that ALDH1A1 mRNA levels gradually drop with culture passages (Fig1D), suggesting that there are multiple pathways for the regulation of gene expression. On the other hand, ALDH1A2 mRNA and protein expression were prominently seen only in HSCs-P1. Next, we transfected HSCs-P1 with non-targeting siRNA or siRNA specific to ALDH1A1 and/or ALDH1A2. siRNA treatment suppressed ALDH1A protein expression (Fig1E) and also reduced levels of all-*trans* RA and 13-*cis*-RA, two RA isomers found in HSCs (Okuno *et al*, 1999), although the relative abundance of isomers varied between different samples (Fig1F). Intriguingly, siRNA treatment also increased autofluorescent lipid droplets (Supplementary Fig S2) and decreased α-SMA levels and cell proliferation rate (Fig1E and Supplementary Fig S3). Thus, suppression of both enzyme activity and expression of ALDH1As led to the inhibition of HSC activation, which agrees with the recent paper that suppression of alcohol dehydrogenase (ADH) 3 inhibits HSC activation (Yi *et al*, 2014). To examine directly whether RA signaling is involved in HSC activation, pre-activated HSCs-d3 were incubated with RAR antagonist (AGN193109) for 3 days and examined for phenotypic changes. RAR antagonist significantly inhibited HSC activation, which were reversed by addition of all-*trans* RA (Fig1G and H). This indicates that RA plays a role in the process of HSC activation.

Phenotypic changes of HSCs by citral. Phase-contrast images (left), oil red O-stained images (middle), and autofluorescence images (right) are shown for HSCs after passage 1 with or without the treatment of citral (100 μM) for 24 h. Scale bar = 10 μm.

Western blot analysis of cell lysates from the citral-treated HSCs in (A). α-tubulin was used as a loading control. The quantitative densitometric data are expressed as the percentage of untreated control and represent the means (SD) for three independent experiments. **P-value = 0.002, paired *t*-test (compared to control).

Protein expression of ALDH1A1 and ALDH1A2 during the culture activation of HSCs. Cell lysates were prepared from HSCs at 3 days after plating (HSCs-d3) and from HSCs after passage 1 (P1), 2 (P2), and 3 (P3), and analyzed by Western blotting.

Real-time PCR analysis for the expression levels of ALDH1A1 and ALDH1A2 during the culture activation of HSCs. The PCR data are expressed as the percentage of HSCs-d3. **P-value, paired *t*-test (*n *= 3) (compared to HSCs-d3), ALDH1A1/HSCs-P3: 0.003, ALDH1A2/HSCs-P1: 0.0018, ALDH1A2/HSCs-P3: 0.001.

Effect of ALDH1A suppression on HSC activation. HSCs-P1 were transfected with non-targeting siRNA or siRNA specific to ALDH1A1 and/or ALDH1A2, and after 48 h, whole-cell lysates were subjected to Western blotting. **P = 0.0023, paired *t*-test (*n *= 3) (siRNA-ALDH1A1/2 compared to control).

Reverse-phase HPLC analysis of whole-cell lysates from the siRNA-ALDH1A-treated HSCs in (E). Typical chromatograms of all-*trans* retinoic acid and 13-*cis* retinoic acid. The retention time is 14 min for 13-*cis*-RA and 23 min for all-*trans* RA.

Phenotypic changes of HSCs by RAR antagonist. HSCs-d3 were incubated with AGN193109 (1 μM) ± all-*trans* RA (10 μM) for 3 days and subjected to Oil red O staining. Scale bar = 10 μm.

Western blot analysis of cell lysates from AGN193109 ± RA-treated HSCs in (G). **P = 0.003, paired *t*-test (*n *= 3) (AGN193109 compared to control).

### Downregulation of RA signaling may contribute to the anti-fibrotic effect of albumin

**Figure 2:**
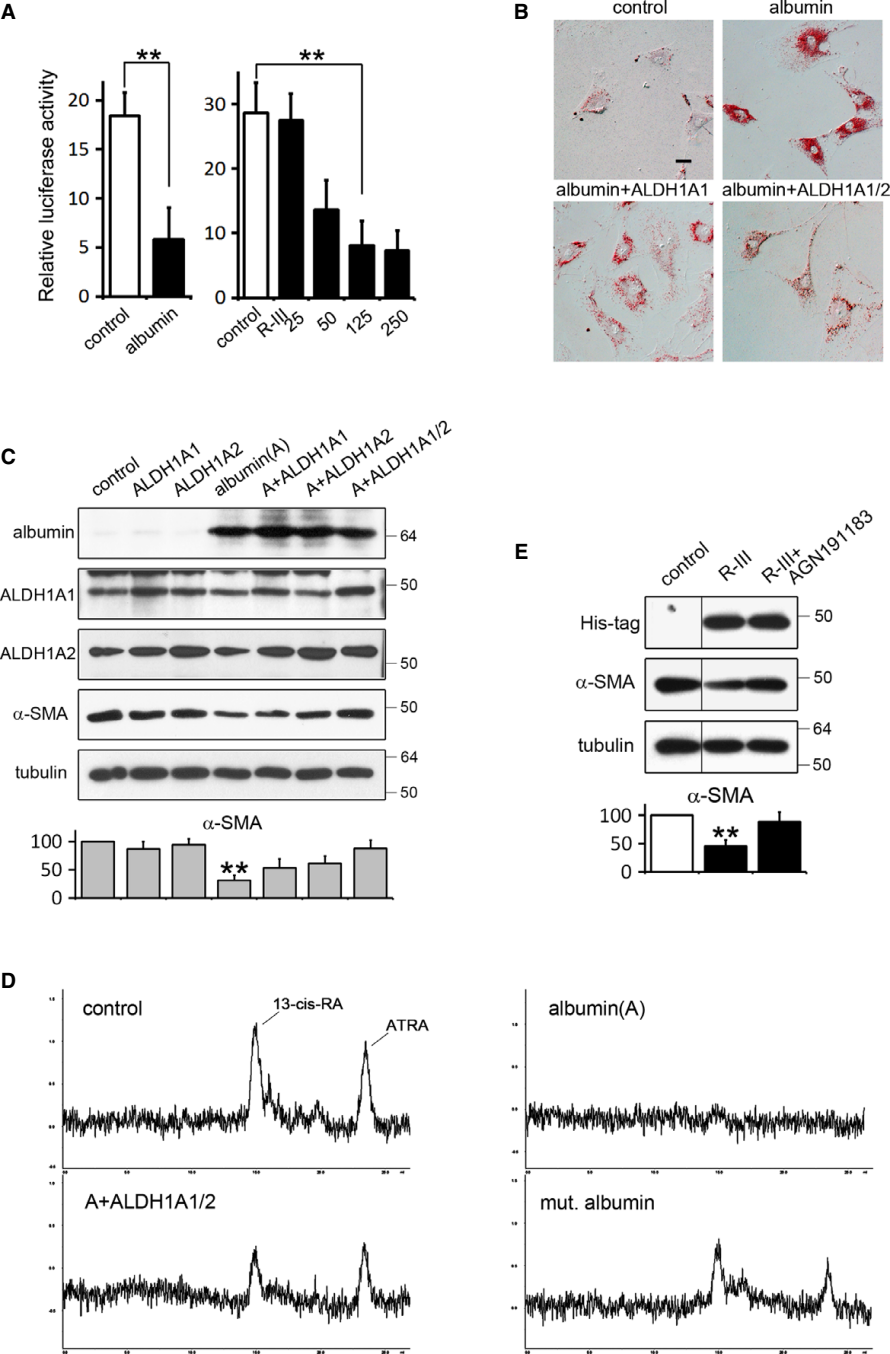
Downregulation of RA signaling may contribute to the anti-fibrotic effect of albumin

We previously showed that albumin expression and R-III (Supplementary Fig S4) treatment is accompanied with a reduction of cellular RA levels (Park *et al*, 2012). To examine whether the anti-fibrotic activity of albumin and R-III is attributed to decreased RA signaling, we carried out several experiments. First, HSCs-P1 were transfected with RARE-luciferase reporter and albumin expression vector. Luciferase assay revealed that albumin expression decreases RA signaling by 70% (Fig2A). A similar effect was observed with R-III treatment in a dose-dependent manner. Secondly, HSCs-P1 were transiently transfected with albumin expression vector in combination with plasmids encoding ALDH1A1 and ALDH1A2 and incubated in the presence of RA for 18 h. Albumin expression inactivated HSCs as previously reported (Kim *et al*, 2009), while co-expression of ALDH1A1 and ALDH1A2 abolished the albumin effect, in addition to increasing cellular RA levels (Fig2B–D). Thirdly, HSCs-P1 were treated with R-III in the presence or absence of RAR agonist (AGN191183) for 18 h. R-III treatment inactivated HSCs with decreased α-SMA levels, but RAR agonist significantly reduced R-III effect (Fig2E). This suggests that the anti-fibrotic effect of albumin and R-III is mediated, at least in part, by downregulation of RA signaling. Lastly, previous reports showed that albumin readily binds to retinoic acid, possibly at fatty acid binding sites (Smith *et al*, 1973; Belatik *et al*, 2012). To examine the possibility that albumin may reduce RA levels (bioavailability) via direct binding, we transfected HSCs-P1 with expression vector for mutant albumin, in which three high-affinity fatty acid binding sites (Arg410, Tyr411, and Lys525), located in domain III, were substituted with an alanine residue (Kragh-Hansen *et al*, 2006). Mutant albumin failed to induce HSC inactivation (Kim *et al*, 2009) and reduced RA levels only partially (Fig2D). Thus, our findings suggest that albumin and R-III downregulate RA levels and its signaling probably via a direct binding, which may contribute to the inactivation of HSCs.

Albumin and R-III downregulate RA signaling. HSCs-P1 were either transfected with RARE Cignal reporter and expression plasmid containing vehicle or albumin (left panel) or treated with R-III (25–250 nM) after transfection with RARE Cignal reporter (right panel). After 36 h, the transfected cells were harvested for luciferase assay. *Renilla* luciferase activity was normalized to firefly luciferase activity. Ratios were normalized against the cells transfected with vehicle plasmid. **P-value, two-sample *t*-test (*n *= 9) (compared to control), albumin: 0.0037, R-III 125 nM: 0.0032.

Phenotypic changes of HSCs by the expression of albumin and/or ALDH1A. HSCs-P1 were transfected with plasmids encoding albumin, ALDH1A1, and ALDH1A2, either individually or in combination, and incubated in the presence of all-*trans* retinoic acid (50 nM) for 18 h. Cells were analyzed by oil red O staining. Scale bar, 20 μm.

Western blot analysis of cell lysates from albumin- and/or ALDH1A-transfected HSCs as in (B). **P = 0.002, paired *t*-test (*n *= 3) (albumin compared to control).

Reverse-phase HPLC analysis of whole-cell lysates from albumin- and/or ALDH1A-transfected HSCs as in (B).

RAR agonist counteracts R-III action. HSCs-P1 were treated with His-tagged R-III (final concentration, 0.15 μM) in the presence or absence of RAR agonist (AGN191183, 1 μM) for 18 h and analyzed by Western blotting. **P = 0.0024, paired *t*-test (*n *= 3) (R-III compared to control). The separating lines demarcate bands that come from non-consecutive lanes of the same gel.

Source data are available online for this figure.

### STRA6 may be involved in the cellular uptake of R-III into HSCs

**Figure 3:**
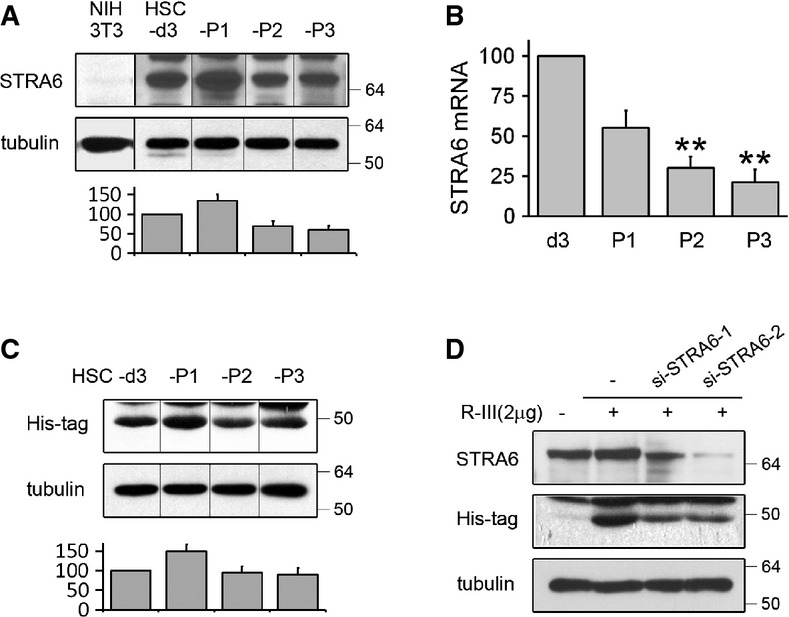
STRA6 may be involved in the cellular uptake of R-III into HSCs

Previous studies suggested that vitamin A bound to retinol-binding protein (RBP) enters HSCs via STRA6 (Kawaguchi *et al*, 2007; Senoo *et al*, 2010), but robust STRA6 expression was not reported in embryonic or adult liver tissue (Bouillet *et al*, 1997; Kawaguchi *et al*, 2007). Thus, we examined the expression of STRA6 in HSCs at different stages of activation in culture by Western blot analysis. STRA6 was expressed in pre-activated and activated HSCs, but relatively higher expression was found in HSCs-P1 (Fig3A). On the other hand, real-time PCR analysis revealed that STRA6 mRNA levels gradually drop with successive culture passages (Fig3B). In a previous study, we showed that R-III enters and inactivates HSCs *in vitro* (Park *et al*, 2012). When HSCs at different stages of activation were incubated with His-tagged R-III for 30 min, Western blotting revealed that levels of R-III uptake paralleled STRA6 protein expression (Fig3C). To test the possibility that STRA6 is involved in R-III uptake, we transfected HSCs-P1 with siRNAs specific to STRA6 and measured the effects. si-RNA treatment suppressed STRA6 expression by > 80% and also markedly reduced R-III uptake (Fig3D).

Protein expression of STRA6 during the culture activation of HSCs. Cell lysates were prepared from HSCs at 3 days after plating (HSCs-3d), and from HSCs after passage 1 (P1), 2 (P2), and 3 (P3), and analyzed by Western blotting. Lysates from NIH3T3 cells were used as a negative control. The separating lines demarcate bands that come from non-consecutive lanes of the same gel.

Real-time PCR analysis for the expression level of STRA6 during the culture activation of HSCs. The PCR data are expressed as the percentage of HSCs-3d. **P-value, paired *t*-test (*n *= 3) (compared to HSCs-d3), HSCs-P2: 0.002, HSCs-P3: 0.0022.

R-III uptake parallels STRA6 level. HSCs at different stages of activation were incubated with His-tagged R-III (final concentration, 0.15 μM) for 30 min, washed, and subjected to Western blotting for R-III uptake.

STRA6-mediated uptake of R-III. HSCs-P1 were transfected with non-targeting siRNA or one of the two different STRA6-specific siRNAs, and after 48 h, cells were incubated with R-III for 30 min and analyzed by Western blotting.

Source data are available online for this figure.

### R-III reduces CCl4-induced liver fibrosis

**Figure 4:**
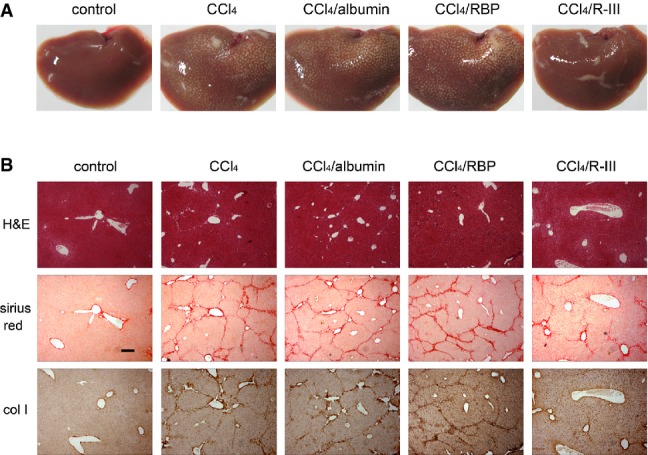
Therapeutic effect of R-III on CCl_4_-induced liver fibrosis

**Figure 5:**
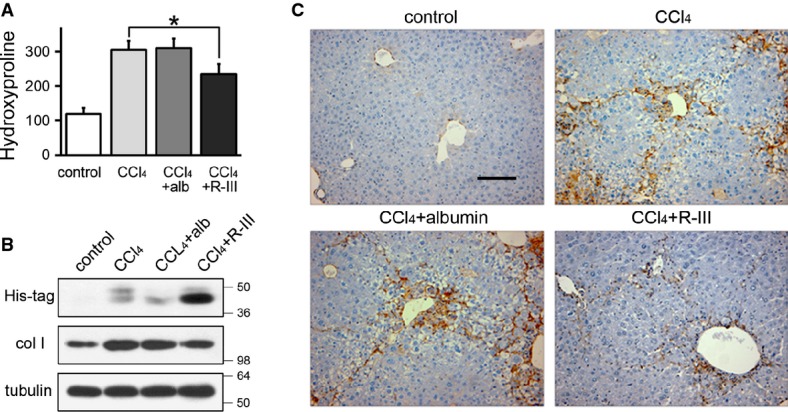
R-III reduces CCl_4_-induced liver fibrosis

**Figure 6:**
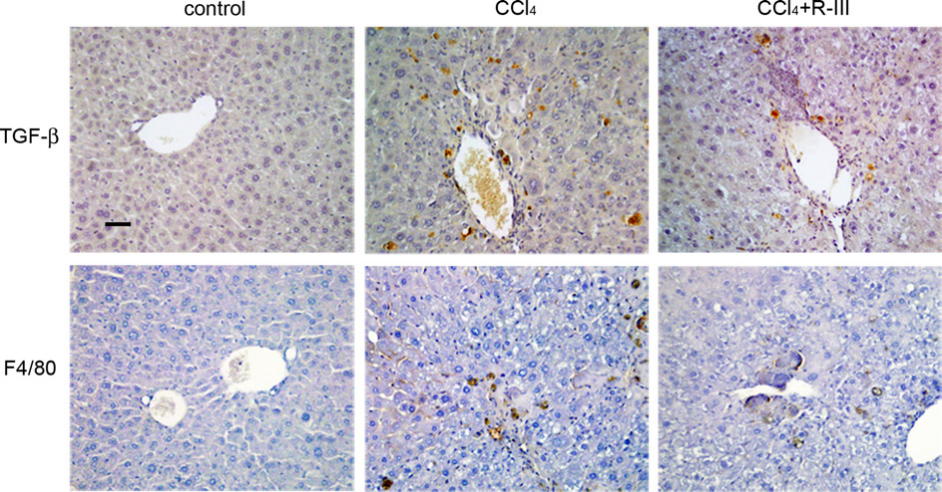
Immunohistochemical staining of liver sections for TGF-β and F4/80 Liver sections from control and CCl_4_- and CCl_4_/R-III-treated mice were immunohistochemically stained for TGF-β and F4/80. Scale bar, 50 μm.

On the basis of the *in vitro* anti-fibrotic activity, we explored the therapeutic effects of R-III on CCl_4_-induced liver fibrosis model. BALB/c mice were treated with CCl_4_ dissolved in mineral oil for 7 weeks and administered via tail vein injection with saline, albumin (10 μg), RBP (5 μg), or His-tagged R-III (10 μg) once per day during the last 2 weeks of CCl_4_ treatment (Supplementary Fig S5). The external surface of the liver in mineral oil/saline-treated control mice was smooth and glistening, while multiple nodules were found macroscopically on the surfaces of livers in CCl_4_/saline-treated mice (Fig4A). Interestingly, R-III treatment significantly reduced nodule incidence, which was not observed in mice treated with either albumin or RBP. The histological analysis of livers in the control mice showed normal architecture, whereas liver fibrosis was severe in CCl_4_/saline-treated mice, as evidenced by disruption of tissue architecture and large fibrous septa formation (Fig4B). Sirius red staining and immunohistochemistry also confirmed extensive collagen deposition in the liver (Fig4B). R-III significantly reduced histopathological alterations and collagen deposition. Hydroxyproline assays showed 35% reduction in collagen content by R-III, which was also confirmed by Western blot (Fig5A and B). Intense immunostaining for α-SMA was found along the fibrotic septa around the central vein in CCl_4_/saline-treated livers, and R-III treatment considerably decreased α-SMA staining (Fig5C), a finding that is consistent with our *in vitro* data indicating that R-III inactivates HSCs. Immunoreactivity with pro-fibrogenic mediator TGF-β was also significantly decreased with R-III treatment (Fig6). Infiltration of F4/80-positive macrophages was increased in CCl_4_/saline-treated livers, whereas R-III treatment reduced F4/80 staining (Fig6).

Representative macroscopic pictures of livers from control and CCl_4_-, CCl_4_/albumin-, CCl_4_/RBP-, and CCl_4_/R-III-treated mice.

Liver sections were stained with H&E and Sirius red, and also subjected to immunohistochemistry for type I collagen. Scale bar, 200 μm.

Hydroxyproline content in the livers from control and CCl_4_-, CCl_4_/albumin-, and CCl_4_/R-III-treated mice (μg/g liver). *P = 0.037, two-sample *t*-test (*n *= 10) (CCl_4_ + R-III compared to CCl_4_-treated mice).

Western blot analysis on liver extracts from the treated mice with antibody against type I collagen.

Immunohistochemical analysis of α-SMA on representative liver sections from the treated mice. Scale bar, 200 μm.

### Intravenously injected R-III localizes in hepatic stellate cells

**Figure 7:**
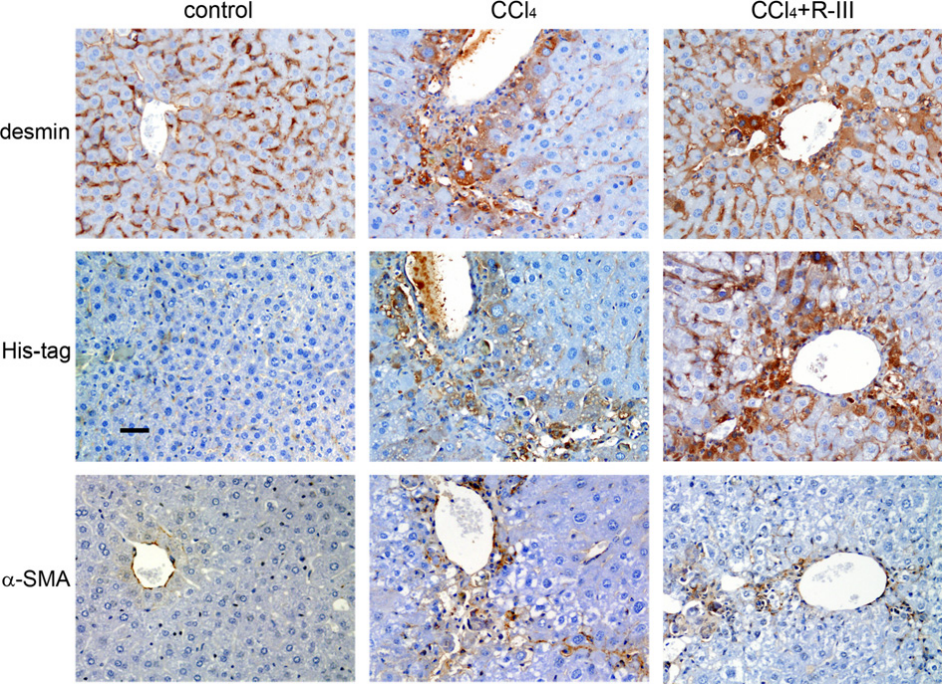
Targeted delivery of R-III to hepatic stellate cells Liver sections from treated mice were immunohistochemically stained for desmin, His-tag, and α-SMA. His-positive signals largely overlapped with desmin/α-SMA staining. Scale bar, 50 μm.

We then investigated the cellular distribution of injected, His-tagged R-III in liver by immunohistochemistry. In normal livers, desmin-positive, quiescent HSCs were distributed in the hepatic parenchyma within the perisinusoidal space, while desmin/α-SMA-positive, activated HSCs were primarily located in septal and portal areas in fibrotic livers (Fig7). Importantly, His-tag staining extensively overlapped with desmin staining, although weak, non-specific signal was also detected in parenchymal cells. This *in vivo* finding, along with early *in vitro* data (Fig3D), suggests that the RBP domain successfully targeted R-III to hepatic stellate cells.

### R-III slows the onset of CCl_4_-induced liver fibrosis

**Figure 8:**
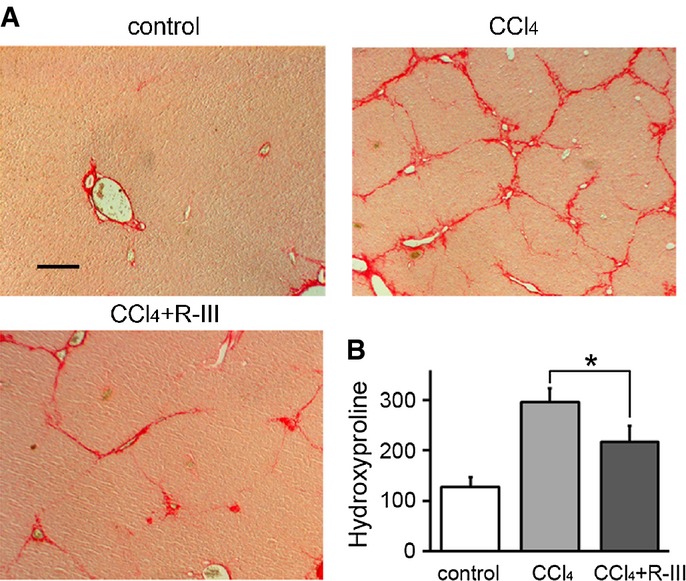
Preventive effect of R-III on CCl_4_-induced liver fibrosis

To examine whether R-III has a preventive effect on CCl_4_-induced liver fibrosis, mice were treated with CCl_4_ and R-III on different days three times per week over a period of 7 weeks (Supplementary Fig S6). Sirius red staining of liver sections showed that R-III treatment markedly reduced collagen deposition (Fig8A). Collagen content was reduced by 45% in the R-III-treated group, as measured using hydroxyproline assays (Fig8B).

Liver sections from control and CCl_4_- and CCl_4_/R-III-treated mice were stained with Sirius red. Scale bar, 200 μm.

Hydroxyproline content in the livers (μg/g liver). *P = 0.034, two-sample *t*-test (*n *= 10) (CCl_4_ + R-III compared to CCl_4_-treated mice).

### R-III reduces BDL-induced liver fibrosis

**Figure 9:**
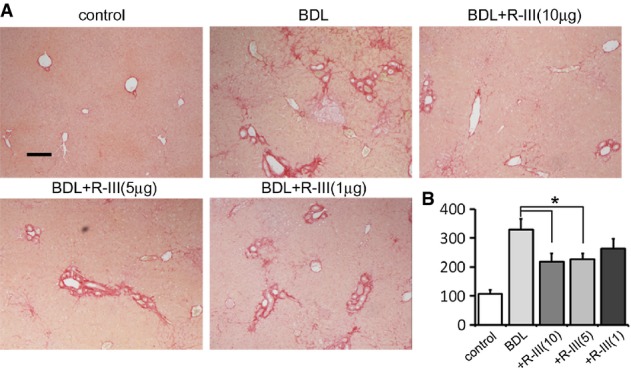
R-III reduces BDL-induced liver fibrosis

Mice underwent BDL and were daily administered with R-III (1, 5 or 10 μg) from 2 to 3 weeks of BDL (Supplementary Fig S7). R-III treatment reduced cholestatic liver fibrosis (Fig9A) and reduced collagen content by up to 45% (Fig9B).

Liver sections from control, BDL, and BDL/R-III-treated mice were stained with Sirius red. Scale bar, 200 μm.

Hydroxyproline content in the livers (μg/g liver). *P-value, two-sample *t*-test (*n *= 10) (compared to BDL-treated mice); BDL + R-III (10 μg): 0.031, BDL+R-III (5 μg): 0.026.

## Discussion

HSCs are considered an attractive target for anti-fibrotic therapies. Several previous reports aimed to inactivate HSCs and reduce liver fibrosis, but no effective therapy for treating liver fibrosis is currently available, mainly due to our incomplete understanding of the molecular mechanism underlying the activation of HSCs and the lack of target specificity of candidate drugs (Ghiassi-Nejad & Friedman, 2008). We have previously showed that forced expression of albumin inactivates stellate cells *in vitro* (Kim *et al*, 2009; Park *et al*, 2012), and developed the recombinant fusion protein R-III, in which albumin domain III is fused to the C-terminus of retinol-binding protein for stellate cell-specific delivery (Choi *et al*, 2012; Park *et al*, 2012). Treatment of HPLC-purified R-III inactivated cultured HSCs as seen by albumin expression.

Our *in vitro* experiment in this study showed that R-III uptake into HSCs is markedly affected by siRNA-STRA6. When injected via tail vein, R-III was predominantly delivered to stellate cells in the liver, indicating that RBP was successfully adopted as a targeting moiety. More importantly, R-III significantly attenuated CCl_4_-induced liver fibrosis with a concomitant reduction of α-SMA-positive cells. This anti-fibrotic effect was not, however, observed with either albumin or RBP (Fig4). R-III also exhibited anti-fibrotic effect on bile duct ligation-induced liver fibrosis. These findings show that R-III is a novel anti-fibrotic drug candidate targeting stellate cells.

Upon stellate cell activation, cytoplasmic lipid droplets collapse and a portion of the retinoid contents are likely released and metabolized. The kinetics of protein expression of ALDH1A1 and ALDH1A2, responsible for retinoic acid (RA) synthesis in HSCs, coincided with RA production; their levels peaked as HSCs freshly isolated from rat liver reached confluence and were passaged (Fig1). The role of retinoids in HSC activation has been proposed, but previous reports about the effects of exogenous retinoids on HSCs and fibrogenesis were contradictory; several studies showed the inhibitory effect of RA on hepatic fibrosis (Wang *et al*, 2007; He *et al*, 2011), while other reports showed the opposite (Okuno *et al*, 1997, 2002). There is no clear answer for this controversy at the moment, although several possible explanations were proposed (Zhou *et al*, 2012). Thus, we sought to evaluate the role of endogenous RA in HSC activation. When enzyme activity or protein expression of ALDH1A1 and ALDH1A2 was suppressed, HSCs underwent cell inactivation with observations of increased lipid droplets and decreases in α-SMA and RA levels. Furthermore, RAR antagonist inhibited HSC activation, indicating that RA signaling plays a role in stellate cell activation.

Albumin binds a wide variety of hydrophobic ligands. Binding of fatty acids to albumin has been extensively studied, and multiple binding sites on albumin have been located (Curry *et al*, 1998; Simard *et al*, 2005; Kragh-Hansen *et al*, 2006). Biophysical studies also showed that albumin readily binds to retinoic acid, possibly at its fatty acid binding sites (Smith *et al*, 1973; Belatik *et al*, 2012). This finding agrees with those of a recent report that albumin sequesters RA, preventing podocyte differentiation (Peired *et al*, 2013). It is also supported by our previous finding that both albumin expression and R-III treatment markedly decrease cellular RA levels in HSCs (Park *et al*, 2012). Thus, it is possible to reason that the anti-fibrotic effect of albumin and R-III is attributed to downregulation of RA signaling which is involved in the process of HSC activation. Our study showed that albumin expression and R-III treatment indeed lead to downregulation of RA signaling in HSCs (Fig2A) and that their anti-fibrotic effects are abolished by ALDH1A overexpression and RA receptor agonist, respectively (Fig2B and E). Furthermore, studies of mutant albumin R410A/Y411A/K525A, which fails to induce HSC inactivation (Kim *et al*, 2009) and modulates RA levels only partially (Fig2D), strongly suggest a direct interaction between RA and albumin (fatty acid binding sites). Therefore, these findings suggest that downregulation of RA signaling by removal of RA may contribute to the anti-fibrotic action of albumin and R-III in HSCs.

Our study showed that there is an increase in cytoplasmic lipid droplets in citral-treated, siRNA-ALDH1A-transfected, and albumin-transfected HSCs-P1, as assessed by oil red O staining. We examined whether retinyl palmitate levels in these inactivated cells were increased by reverse-phase HPLC but found no significant differences. The reason may be that the detection method is not sensitive enough to detect small change. In our experiments, retinol and palmitate were not added to the culture media. Alternatively, the reappearance of lipid droplets may not accompany new synthesis of retinyl esters. At present, we have no clear answer for this. There was, however, an increase in autofluorescence (Fig1A and Supplementary Fig S2), indicating that lipid droplets likely contain retinoids. Further studies are needed to elucidate the detailed mechanisms of albumin/R-III action and HSC activation.

The HSC population in a healthy, uninjured liver shows heterogeneity in levels of HSC activation markers and retinyl ester (D'Ambrosio *et al*, 2011). Thus, it is likely that one subset of HSCs in normal liver may have elevated STRA6 levels and be ready for vitamin A uptake, while the majority of HSCs have abundant perinuclear lipid droplets and may not need to maintain high STRA6 level. Interestingly, it was observed that STRA6 protein level was not correlated with mRNA level in HSCs at day 3 after plating (pre-activated HSCs) (Fig3A and B). Although further study is required to elucidate the detailed mechanisms underlying these phenomena, protein degradation is a likely explanation.

Retinoid-storing stellate cells also exist in extrahepatic organs such as pancreas, kidney, spleen, intestine, and lung (Nagy *et al*, 1997). These cells show striking similarities in morphology and perivascular location, which suggests that activated stellate cells may contribute to the myofibroblast cells seen in the fibrotic extrahepatic tissues (Erkan *et al*, 2012). As intravenously injected R-III is detected also in extrahepatic organs such as brain, lung, spleen, pancreas, kidney, and intestine (Choi *et al*, 2012), we have examined whether R-III reduces renal and pulmonary fibrosis (manuscript in preparation). We also found that R-III has a plasma half-life of ∽20 h (Supplementary Fig S8) and that mice showed no apparent side effects after receiving R-III (10 μg, intravenous injection once daily for 7 days).

In this study, we demonstrated that the fusion protein R-III alleviated CCl_4_- and bile duct ligation-induced liver fibrosis by inducing HSC inactivation. Our finding may provide a new approach to treating fibrotic diseases in different tissues.

## Materials and Methods

### Animals

Male Sprague-Dawley rats of 6–8 weeks of age and male BALB/c mice of 6–8 weeks were purchased from Orient (Charles River Korea, Seoul, Korea). Animals were fed a commercial diet R03-10 (Safe, Augy, France) and maintained under temperature-, humidity-, and light-controlled conditions. Animal experiments were approved by our institutional review board and complied with the Guide for the Care and Use of Laboratory Animals.

### Hepatic stellate cell isolation and culture

Rats were anesthetized intraperitoneally with sodium thiopental, and primary HSCs were isolated as described previously (Langer *et al*, 2008) and cultured in DMEM supplemented with 10% fetal bovine serum. The purity of stellate cells was > 90% as assessed by the presence of cytoplasmic lipid droplets and Western blotting using anti-tyrosine aminotransferase antibody. The isolated cells reach confluence after 5–7 days in culture and subcultured in a threefold dilution.

### Western blot analysis

Electrophoresis and immunoblotting were performed as described (Kim *et al*, 2009). Primary antibodies used were STRA6 (Prosci #46-715, Poway, CA, USA), α-SMA (Sigma-Aldrich #A2547, St. Louis, MO, USA), albumin (Bethyl Laboratories #A110-134A, Montgomery, TX, USA), His-tag (AB Frontier #LF-MA-20209 Seoul, Korea), type I collagen (Calbiochem #234168, San Diego, CA, USA), ALDH1A1 (Abcam #ab52492, Cambridge, UK), ALDH1A2 (Abcam #ab156019), and α-tubulin (Cell Signaling Technology #2125, Beverly, MA, USA).

### Analysis of gene expression using real-time PCR

cDNA was synthesized from total RNA, and real-time PCR was performed using LightCycler-FastStart DNA Master SYBR Green 1 (Roche, Mannheim, Germany) with gene-specific primers (Supplementary Table S1). To control for variations in the reactions, PCR products were normalized against glyceraldehyde 3-phosphate dehydrogenase (GAPDH) expression.

### Purification of (His)6-tagged R-III fusion protein

Expression vector for mouse R-III and the high producing, stably transfected 293 cell lines were prepared as described previously (Choi *et al*, 2012). For the purification of R-III, conditioned medium was prepared from transfected 293 cells grown in serum-free medium for 4 days, fractionated with ammonium sulfate (55%), and then subjected to a His Trap affinity column. The sample was further purified using Resource Q. Purified protein was dialyzed against deionized water, freeze-dried, and dissolved in saline solution. The purity of R-III is > 95%, as determined by SDS–PAGE and protein staining.

### Transfection and si-RNAs

HSCs were transiently transfected using Lipofectamine 2000 (Invitrogen, Carlsbad, CA, USA) and analyzed after 24 h. Knockdown of STRA6, ALDH1A1, or ALDH1A2 was performed using specific siRNA duplexes sets (Supplementary Table S2) from Bioneer (Daejeon, Korea).

### Luciferase assay

HSCs were transiently transfected with a combination of RARE Cignal report (Qiagen, Germantown, MD, USA), including inducible transcription factor responsive construct and constitutively expressing *Renilla* luciferase construct, and the respective vector (pcDNA3.1 or pcDNA3.1-albumin). After 36 h, the luciferase assay was performed using Dual Luciferase Reporter Assay System (Promega, Madison, WI, USA) according to the manufacturer's instructions. The ratio between firefly and *Renilla* luciferase was used to normalize the transfection efficiency. Quantification was performed on three independent experiments executed in triplicate.

### Liver fibrosis induced by CCl_4_ or BDL

For CCl4-induced liver fibrosis study, BALB/c mice were treated with CCl_4_ (1 ml/kg body weight; 1:1 dilution with mineral oil) or mineral oil as a control by intraperitoneal (i.p.) injection three times per week for 7 weeks. For the determination of therapeutic effects of R-III, a total of 20 CCl_4_-treated mice were randomly divided into four groups (each group = 5 mice); mice were administered via tail vein injection (without anesthesia) with saline, albumin (10 μg), RBP (5 μg), or R-III (10 μg) every day during the last 2 weeks of CCl_4_ treatment (Supplementary Fig S5). For the assessment of preventive effects of R-III, a total of 10 CCl_4_-treated mice were randomly divided into two groups (five mice per group) and administered with saline or R-III (Supplementary Fig S6). Three to five mineral/saline-treated mice were used as normal controls for each experiment. For the study of liver fibrosis induced by BDL, mice were anesthetized intraperitoneally by ketamine and xylazine. After midline laparotomy, the common bile duct was double-ligated and transected between the ligatures. The sham operation was performed similarly without BDL. After BDL for 7 days, R-III (0, 1, 5, or 10 μg) was daily administered for another 2 weeks (five mice per group) (Supplementary Fig S7). All experiments were repeated twice.

### Immunohistochemical analysis

After routine processing, sections (5 μm thick) of formalin-fixed, paraffin-embedded liver tissues were prepared and stained with H&E for histological analysis or Sirius red for collagen deposition. Tissue sections were also stained immunohistochemically with the following antibodies: desmin (Dako #M0760, Carpinteria, CA, USA), α-SMA (Abcam #ab32575), His-tag (Abcam #ab84162), TGF-β (Santa Cruz #sc146, Santa Cruz, CA, USA), and F4/80 (Serotec #MCA497, Kidlington, UK).

### Hydroxyproline measurement

Liver hydroxyproline levels were measured following the manufacturer's protocol (Sigma-Aldrich). Briefly, liver tissue was homogenized in distilled water and mixed with an equal volume of concentrated hydrochloric acid (∽12 N HCl), and the homogenates were incubated at 120°C for 3 h. After hydrolysis, the samples were oxidized with Chloramine T (Sigma-Aldrich), followed by enzymatic reaction with 4-dimethylaminobenzaldehyde (DMAB) solution. Sample absorbance was measured at 560 nm in duplicate. Hydroxyproline content was expressed at nanogram of hydroxyproline per milligram liver.

### Oil red O staining and autofluorescence

HSCs were stained with Oil red O to visualize lipid droplets, essentially as described previously (Kim *et al*, 2009). Oil red O was diluted in triethyl phosphate instead of isopropanol. The fast-fading vitamin A-specific autofluorescence was observed with light of 330–360 nm (UV) laser.

### RP-HPLC

Cells were quantified and extracted as described (Merris *et al*, 2002). Reverse-phase chromatography (RP-HPLC) was carried out on an AKTA Explorer HPLC system (GE Healthcare Life Sciences, Piscataway, NJ, USA). The chromatographic conditions were as previously described (Radaeva *et al*, 2007).

### Statistical analysis

Results are expressed as mean ± standard deviation (SD). Paired *t*-test or two-sample *t*-test were performed where appropriate. Comparisons were considered significant at *P* < 0.05.

## Supporting Information

Supplementary Figures and Tables

Review Process File
