# Supplementary material for: Fusion protein of retinol-binding protein and albumin domain III reduces liver fibrosis
Source: EMBO Mol Med. 2015 Apr 11;7(6):819–30. doi: 10.15252/emmm.201404527 (PMC4459820; doi:10.15252/emmm.201404527)
Supplement: Supplementary file 1 [file emmm0007-0819-sd1.pdf]

Table of contents

page 2~3: Supplementary figure legend

page 4~11: Supplementary figures 1~8

page 12: Supplementary table legend

page 13~14: Supplementary tables 1~2

Supplementary Fig. 1. Hepatic stellate cells were isolated from rat liver and cultured on plastic dish. Liver tissue extracts and cell lysates from HSCs after passage 1 and 2 were analyzed for heptatocyte-specific marker tyrosine aminotransferase by Western blotting analysis.

Supplementary Fig. 2. Increase in autofluorescent lipid droplets by siRNA-mediated knockdown of ALDH1A. HSCs after passage 1 were transfected with siRNA specific to either ALDH1A1 or ALDH1A2 or both, and after 48 h cells were subject to oil red O staining (left panel) or exposed to UV light (330 nm, right panel). Scale bar = 10 μm

Supplementary Fig. 3. Reduced cell proliferation rate by siRNA-mediated knockdown of ALDH1A. HSCs after passage 1 were transfected with siRNA specific to either ALDH1A1 or ALDH1A2 or both, and after 48 h cell numbers were counted. **$p<0.01$ compared with control.

Supplementary Fig. 4. Schematic diagram of RBP-albumin (III) fusion proteins R-III, compared to albumin and RBP.

Supplementary Fig. 5. Schedules for the treatment of BALB/c mice with CCl_4, albumin, RBP and R-III are described. Mice were injected intraperitoneally (i.p.) with CCl_4 (*black arrowheads*) and were also administered intravenously (i.v.) with saline alone, albumin (10 μg), RBP (5 μg) or R-III (10 μg) dissolved in 0.1 ml saline daily during the last 2 weeks in CCl_4 treatment (*hatched box, grey arrows*).

Supplementary Fig. 6. Schedules for the treatment of BALB/c mice with CCl_4 and R-III are described. Mice were injected with CCl_4 (*black arrowheads*) and [R-III (10 μg) or saline alone] (*gray arrows*) three times per week for 7 weeks.

Supplementary Fig. 7. Schedules for the treatment of BALB/c mice with BDL and R-III are described. Mice underwent BDL (*black arrowheads*) and were administered intravenously (i.v.) with saline or R-III (1, 5 or 10μg) dissolved in 0.1 ml saline daily from 2 to 3 weeks of BDL (*hatched box, grey arrows*).

Supplementary Fig. 8. Mice (n=4) were injected with His-tagged R-III (5μg) via tail vein, and blood was collected from facial vein at the indicated time. Equivalent amounts of plasma protein were analyzed by Western blotting using anti-His tag antibody and the intensity of bands was quantified by densitometry and plotted against time.

Figure S1

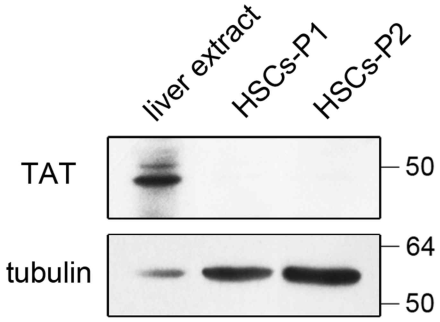

Figure S2

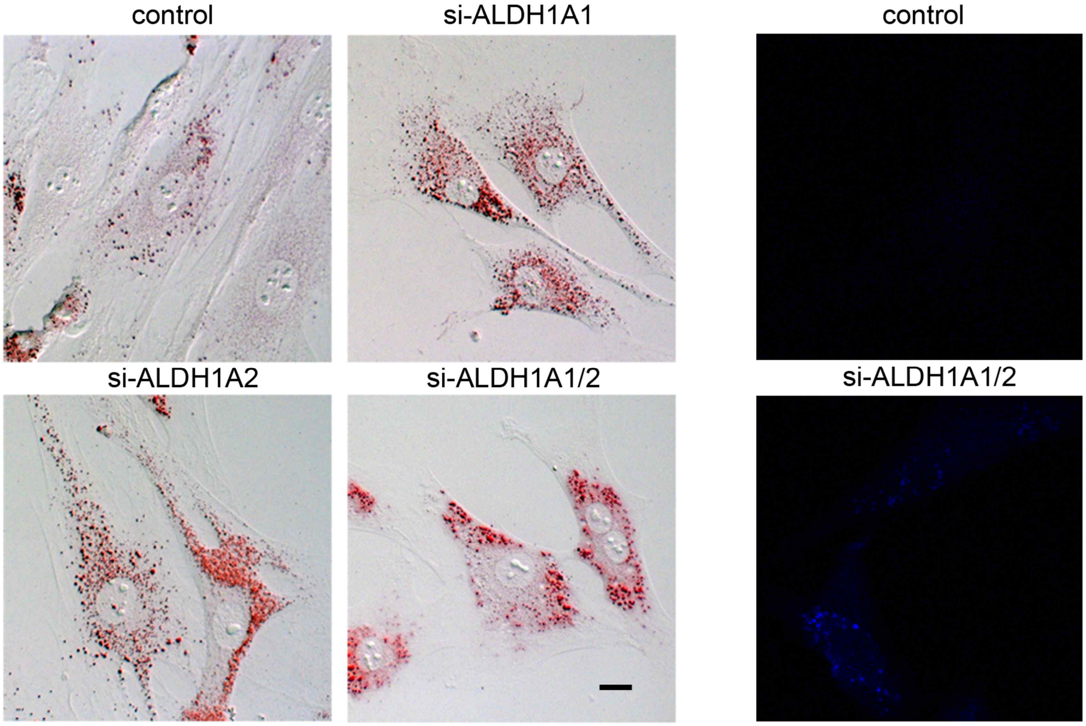
control
si-ALDH1A1
control
si-ALDH1A2
si-ALDH1A1/2
si-ALDH1A1/2

Figure S3

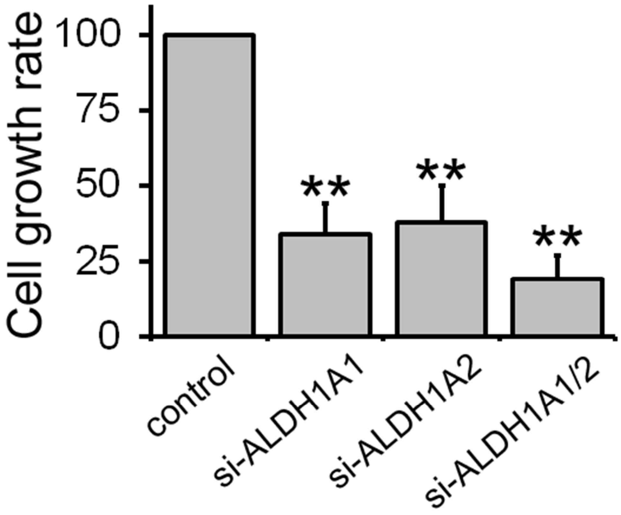

Cell growth rate
100
75
50
25
0
**
**
**
control
si-ALDH1A1
si-ALDH1A2
si-ALDH1A1/2

Figure S4

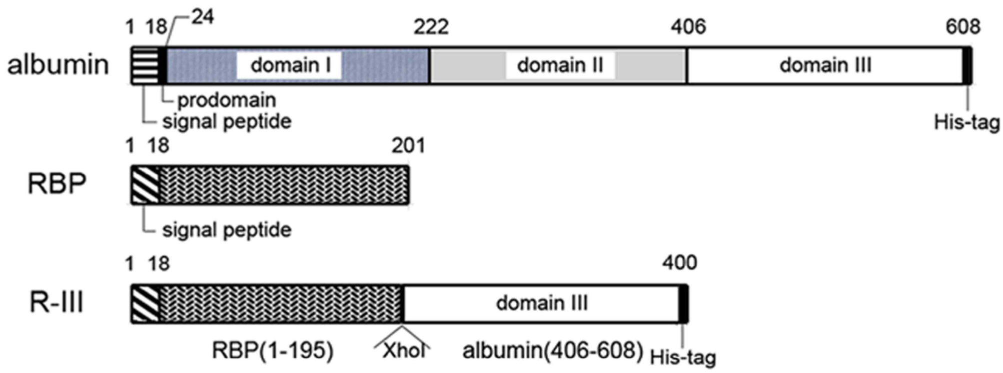
1
18
24
222
406
608
albumin
domain I
domain II
domain III
prodomain
signal peptide
His-tag
1
18
201
RBP
signal peptide
1
18
400
R-III
domain III
RBP(1-195)
XhoI
albumin(406-608)
His-tag

Figure S5

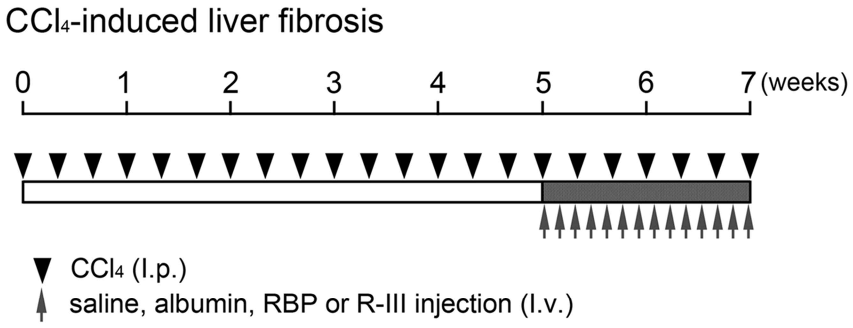
CCl4-induced liver fibrosis
0
1
2
3
4
5
6
7 (weeks)
CCl4 (I.p.)
saline, albumin, RBP or R-III injection (I.v.)

Figure S6

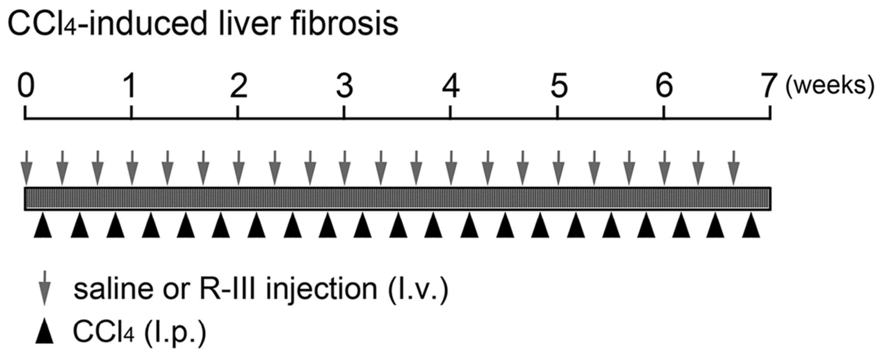
CCl4-induced liver fibrosis
0
1
2
3
4
5
6
7
(weeks)
saline or R-III injection (I.v.)
CCl4 (I.p.)

Figure S7

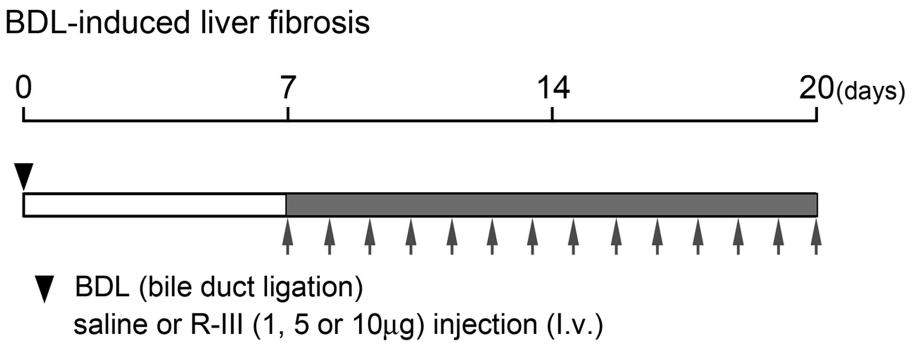
BDL-induced liver fibrosis
0
7
14
20(days)
BDL (bile duct ligation)
saline or R-III (1, 5 or 10μg) injection (I.v.)

Figure S8

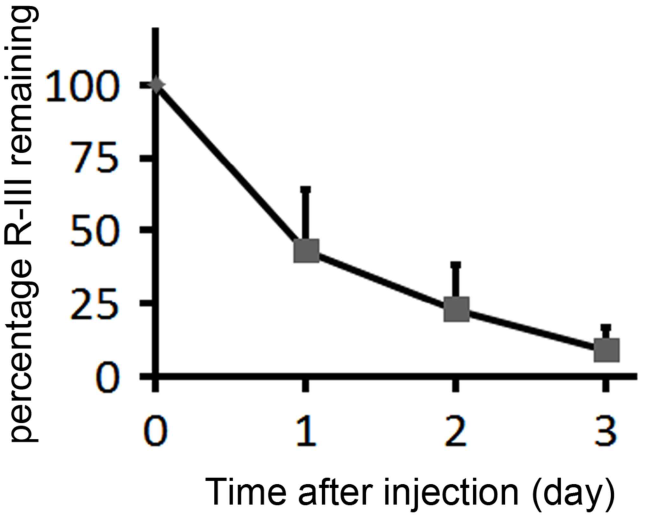

percentage R-III remaining
100
75
50
25
0
0
1
2
3
Time after injection (day)

Supplementary Table 1. Primers used for real-time PCR.

Supplementary Table 2. Sequences of sense and antisense primers used in si-RNA experiments.

Supplementary Table 1. Primers used for real-time PCR.

Gene	forward primer	reverse primer
Stra6	5'-TGC TGG ACT CTG GAG ATG-3'	5'-GTG ATC ACC TGC CCA TC-3'
Raldh1	5'-GCC GAC TTG GAC ATT GCT-3'	5'-GCT CGC TCA ACA CTC TTT CTC-3'
Raldh2	5'-TCA GAG AGT GGG AGA GTG TT-3'	5'-CAC AGA ACC AAG AGA GAA GG-3'
GAPDH	5'-GAA CGG GAA GCT CAC TGG C-3'	5'-GCA TGT CAG ATC CAC AAC GG-3'

Supplementary Table 2. Sequences of sense and antisense primers used in si-RNA experiments.

Gene	sense	anti-sense
Stra6-I	GUGCUUGUCCACAAGACUA	UAGUCUUGUGGACAAGCAC
Stra6-II	GUCUACAUCCUCCCUCUCA	UGAGAGGGAGGAUGUAGAC
Raldh1	CUACAAUAGAGGCCAUCAA	UUGAUGGCCUCUAUUGUAG
Raldh2	CAGACAAGGUGGAUAUAGA	UCUAUAUCCACCUUGUCUG
