# Supplementary material for: Fusion protein of retinol-binding protein and albumin domain III reduces liver fibrosis
Source: EMBO Mol Med. 2015 Apr 11;7(6):819–30. doi: 10.15252/emmm.201404527 (PMC4459820; doi:10.15252/emmm.201404527)
Supplement: Supplementary file 2 [file emmm0007-0819-sd2.pdf]

EMBO Molecular Medicine

Fusion protein of retinol-binding protein and albumin domain III reduces liver fibrosis

Hongsik Lee, Hyeyeun Jeong, Sangeun Park, Wonbaek Yoo, Soyoung Choi, Kyungmin Choi, Min-Goo Lee, Mihwa Lee, DaeRyong Cha, Young-Sik Kim, Jeeyoung Han, Won-Kon Kim, Sun-Hwa Park and Junseo Oh

Corresponding author: Junseo Oh, Korea University, College of Medicine

Review timeline:

Submission date:	11 August 2014
Editorial Decision:	19 September 2014
Revision received:	09 January 2015
Editorial Decision:	17 February 2015
Revision received:	03 March 2015
Accepted:	09 March 2015

Transaction Report:

(Note: With the exception of the correction of typographical or spelling errors that could be a source of ambiguity, letters and reports are not edited. The original formatting of letters and referee reports may not be reflected in this compilation.)

Editor: Roberto Buccione

1st Editorial Decision 19 September 2014

Thank you for the submission of your manuscript to EMBO Molecular Medicine.

In this case we experienced unusual difficulties in securing three willing and appropriate reviewers, in part due to the overlap with the vacation season. As a further delay cannot be justified I have decided to proceed based on the two available evaluations.

As you will see, although the Reviewers appear to have opposing sentiments on your manuscript, the issues raised, although significant, are consistent.

The main, shared concern is that additional experimentation on an alternative non-toxic model of liver fibrosis (inflammatory or viral) is necessary to confirm the main finding showing that HSC-retargeted albumin ameliorates liver fibrosis.

Reviewer 1, in addition to the above, also lists several concerns that require your action including, but not limited to several instances of improper or missing citations and contextualisation of prior work, many missing fundamental experimental details and controls, lack of clear evidence that ATRA can rescue the activated phenotype, lack of clear support for the suggestion that R-III uptake is mediated by STRA6.

Reviewer 2 also mentions the need to verify the dose-dependence of the effects of R-III and asks about the very important issue, in a true translational perspective, of the potential side effects of R-III administration.

I should also mention that since the main message of your work is a translational rather than conceptual one, I am sure you will understand that to fully address the issues raised by the Reviewers is of prime importance and that without these improvements we will not be able to move forward with your manuscript.

In conclusion, while publication of the paper cannot be considered at this stage, given the potential interest of your findings, we are prepared to consider a substantially revised submission, with the understanding that the Reviewers' concerns must be addressed with additional experimental data where appropriate and that acceptance of the manuscript will entail a second round of review. This also includes significantly upgrading the significance of your findings by extending experimentation to an alternative animal model of liver fibrosis.

Please note that it is EMBO Molecular Medicine policy to allow a single round of revision only and that, therefore, acceptance or rejection of the manuscript will depend on the completeness of your responses included in the next, final version of the manuscript.

As you know, EMBO Molecular Medicine has a "scooping protection" policy, whereby similar findings that are published by others during review or revision are not a criterion for rejection. I understand that in this case, to address the above might entail a significant amount of additional work and time and might be technically challenging. However, I do ask you to get in touch with us after three months if you have not completed your revision, to update us on the status. Please also contact us as soon as possible if similar work is published elsewhere.

I look forward to seeing a revised form of your manuscript in due time.

***** Reviewer's comments *****

Referee #1 (Comments on Novelty/Model System):

Quality of micrographs is generally good. However additional controls are needed and/or additional information is needed for the work to be reproduced.

The model system is adequate. It would be interesting to confirm this with another, more relevant model of hepatic fibrosis, such as a viral infection model.

Referee #1 (Remarks):

This manuscript presents some data that are exciting and relatively convincing, but it is weakened by some misstatements, some overstatements, and a lack of certain controls and comparisons, as noted below. These should be addressed to make a stronger manuscript. There are also experimental details that must be added for successful reproduction of the studies.

Introduction, page 3: The scenario is given as "Vitamin A (retinol), acquired from the diet, is bound to retinol-binding protein 4 (RBP4) in the bloodstream, and is taken up initially by liver cells and transferred to HSCs via RBP receptor STRA6 (Kawaguchi et al, 2007; Senoo et al, 2010)." -This sentence is incorrect. It fails to point out that the transporter of vitamin A from the gut to the liver is the chylomicron, which as a chylomicron remnant is taken up by the hepatocytes, and that STRA6 expression is very low if not nil in the liver. Some research supports a role of RBP4 in transferring retinol from the parenchymal cell to the HSC (see papers by Senoo H, and others) but this is not what the introduction says. This statement and its supporting references need to be corrected.

The statement on pages 3-4 including "effects of retinoids, including all-trans- and 9-cis- and 13-cis retinoic acid, are primarily mediated by two families of nuclear receptors, retinoic acid receptor (RAR) and retinoid X receptor (RXR) (Balmer & Blomhoff, 2002)" is partially incorrect. The action of 13-cis RA has never been shown to be mediated by RARs, and 9-cis-RA is now dubious as a physiological ligand of the RXRs. The statement and references need to be corrected and updated.

Results, Page 4. "The effects of exogenous retinoic acid (RA) on HSCs or liver fibrosis are controversial; several studies showed that RA inactivated HSCs and alleviated hepatic fibrosis,

while other reports showed the opposite (Friedman, 2008; Zhou et al, 2012)." --Please place the references immediately after the positions they support so that it is clear which shows which effect. If these are reviews, please use primary references to support. (Also on page 10.)

"HSCs after passage 1 (HSCs-P1; activated HSCs) were incubated with citral, an inhibitor of retinaldehyde dehydrogenases (RALDH) that irreversibly convert retinaldehyde to RA, and examined for changes. Citral treatment increased cytoplasmic lipid droplets (Fig. 1A), as assessed by Oil red O staining, and decreased a-SMA levels (Fig. 1B)..," --The cells should be extracted and tested for their content of retinyl esters. Citral, as a lipid aldehyde, is not just specific for inhibition of RALDHs, and it could be that the droplets are lipids and therefore they stain with Oil Red O, but they might not necessarily be composed of retinyl esters. This should be confirmed biochemically. The methods and legend also need to clarify how much retinol was present in the medium as a source of retinyl ester formation. If there are really increased retinyl esters in the HSC cells then there should be a commensurate reduction in retinol to account for this.

Page 5, "Intriguingly, siRNA treatment (RALDH1 and -2) also increased lipid droplets (Fig. S2) and decreased a-SMA levels and cell proliferation rate (Fig. 1E and S3)." -- Again, the implication is that this is due to differences in retinoids but the investigators need to assure their readers that it is not due to differences in other lipids. The statement that follows which says this "agrees with the recent paper that suppression of alcohol dehydrogenase (ADH) 3 inhibits HSC activation (Yi et al, 2014)." actually would support any lipid alcohol as being involved, as these classes of enzymes generally metabolize more than one substrate. The use of AGN193109 as a retinoid signaling antagonist is reassuring but there is no experiment showing that all-trans-RA can "rescue" the activated phenotype. This should be added.

Page 6, "Luciferase assay revealed that albumin expression decreases RA signaling by 70% (Fig. 2A)." --This is interesting but the statement should include the concentration of RA that was added and, if possible, the molar ratio of albumin to RA in the cells. It is not possible to infer whether this is due to the binding and therefore the effective removal of RA by albumin or to another effect.

Fig 2B: 5μM RA was used. Were lower concentrations also tested? 5 μM is far above the plasma level of, e.g, 10-50 nM. The relevance of this in vitro experiment would be greater if a more physiological concentration was tested and shown to be effective.

"...while co-expression of RALDH1 and -2 abolished the albumin effect, in addition to increasing cellular RA levels (Fig. 2D)." As above, as the RALDHs need substrate to produce RA, the text should specify how much retinol was in the medium and how much retinyl ester the HSCs-P1 contained. As the activation process results in the loss of retinyl esters, the longer-cultured primary HSCs might not be able to produce RA efficiently. Have the authors conducted similar experiments with activated HSCs?

"This suggests that the anti-fibrotic effect of albumin and R-III is largely mediated by downregulation of RA signaling." Since the HSC-P1 cell stage is most likely prefibrotic, it may be an earlier event that is being regulated.

"Lastly, previous reports showed that albumin readily binds to retinoic acid, possibly at fatty acid binding sites (Belatik et al, 2012)." The binding of RA to albumin was shown decades ago and that reference should be added (Smith et al, J Biochem 1973).

Page 7, "Mutant albumin failed to induce HSC inactivation (Kim et al, 2009) and reduced RA levels only partially (Fig. 2D)." This is a good experiment and helps to build support for the concept.

"Cellular uptake of R-III into HSCs is STRA6-dependent" (Fig. 3) - -This section focuses on STRA6, which, as the authors have noted, is not "robustly" expressed in the liver. But the RBP4-R2 receptor described by Alapatt et al (J Biol Chem 2013) might be a better candidate. The RNA collected from these studies should be examined for the expression of this alternative receptor for RBP4. The discussion actually states that the uptake of R-III was "mediated" by STRA6. This was never shown and the statement is too strong. A better model would be to use cells from liver of STRA6 knock-out mice. The authors should add experiments or modify their statement.

Page 8, The studies using R-III in vivo are quite interesting and the difference in the appearance of the livers is quite striking. Do the investigators know the half-life of R-III in mouse plasma? Since it was delivered once per day, do we know whether more frequent or less frequent delivery is more or less effective?

"In normal livers, desmin-positive, quiescent HSCs were distributed in
the hepatic parenchyma within the perisinusoidal space, while desmin/a-SMA-positive,
activated HSCs were primarily located in septal and portal areas in fibrotic livers (Fig. 6)." -- In Fig. 6, the distributions of desmin and a-SMA actually look rather different. Do they both represent HSCs? Why isn't the overlap closer?

It is stated that paired statistical analysis was performed. What were the pairs? It looks like the mouse study must have used groups of mice, not pairs. For some data, such as shown in Fig. 1D, 3B, S3, no statistics are shown. Please include.

Other comments--
Information that is necessary in order for the study to be reproduced by others: Sex of mice; type and source of the diet fed; method and agent of euthanasia; length of culture before each passaging of HSC; composition of the HSC culture medium.

Page 12: It is stated that "the purity of HSC was assessed by lipid droplets...", which to some extent wouldn't they also be present in parenchymal cells? What controls were used to rule out or quantify parenchymal cell contamination of the HSC?

What STRA6 antibody was it? The specificity of anti-STRA6 #26-445 from ProSci is listed as H (human), not rat or mouse. Was this the antibody that was used? Likewise, the catalogue numbers of the other antibodies should be listed. Negative staining controls should also be described.

"intravenously administered with saline, albumin, RBP or His-tagged
R-III once per day during the last 2 weeks of CCl4 treatment." (also page 14). Please specify the site of i.v. injection and whether anesthesia was used. If so, was there a separate anesthesia control group or were the control mice injected with a placebo by the same route?

Minor points needing correction: please search for and correct--

Real-time RCR analysis

Grammar: suggesting that there are multiple regulation of gene expression

RA and ATRA and all-trans retinoic acid are used interchangeably-all-trans-RA with simply RA as the abbreviation is the preferred term.

Referee #2 (Comments on Novelty/Model System):

CCl4 represent a model of toxic liver injury. Other, more inflammatory models should also be tested.

Referee #2 (Remarks):

Liver fibrosis and its end-stage, liver cirrhosis represent a common response to chronic liver injury and are characterized by excessive production and deposition of extracellular matrix (ECM) components. Presently, despite enormous research activities, no effective chemoprevention or therapy for liver fibrosis and (except for the removal of the causative agent) is established, thus underscoring the need for innovative approaches in the field.
Activated hepatic stellate cells (HSCs) are the major source of extracellular matrix during liver fibrosis and thus represent the most obvious target for antifibrotic drugs. Recently, the group providing the present manuscript demonstrated that overexpression of albumin in HSC inhibits HSC

activation a process which requires its intact fatty acid-binding sites in domain III. Moreover, they published a recombinant fusion protein named "R-III", in which albumin domain III was fused to the C-terminus of Retinol binding protein (RBP) 4, for efficient stellate cell-targeting delivery. While the basal characteristics of this fusion-protein as well as its inhibitory effect on HSC activation (in vitro) were already published in 2012, the authors provide now evidence for a potential therapeutic use against liver fibrosis in CCL4 induced murine liver fibrosis. Interestingly, the authors explain this effect by a down-regulation of RA signalling.

While being interesting and technically well performed, to my opinion some questions remain open:
1) Is the effect of R-III dose dependent ?
2) The authors show an enrichment of R-III in HSC within the liver, is R-III also observed in other organs?
2) Are there any side effects of R-III treatment?
3) Does the inhibition of stellate cell activation also inhibit inflammatory processes in liver fibrosis e.g. infiltration of the liver by immune cells?
4) Is there any effect on classical fibrotic pathways such as the TGF-beta/ SMAD pathway?
5) Is the effect specific for toxic models of liver fibrosis, which features only some of the complex aspects driving liver fibrosis in the human, thus can the results on liver fibrosis be reproduced e.g. in metabolic and more inflammatory models of liver fibrosis?

1st Revision - authors' response 09 January 2015

Response to reviewer comments

Referee #1

The model system is adequate. It would be interesting to confirm this with another, more relevant model of hepatic fibrosis, such as a viral infection model.

We agree with the reviewer's opinion and have examined the therapeutic potential of R-III using bile duct ligation model of hepatic fibrosis. Administration of R-III attenuated cholestatic liver fibrosis and reduced collagen content by up to ~50%. Results from animal study are included in the revised manuscript (page 9 and Fig. 9).

Introduction, page 3: The scenario is given as "Vitamin A (retinol), acquired from the diet, is bound to retinol-binding protein 4 (RBP4) in the bloodstream, and is taken up initially by liver cells and transferred to HSCs via RBP receptor STRA6 (Kawaguchi et al, 2007; Senoo et al, 2010)." -This sentence is incorrect. It fails to point out that the transporter of vitamin A from the gut to the liver is the chylomicron, which as a chylomicron remnant is taken up by the hepatocytes, and that STRA6 expression is very low if not nil in the liver. Some research supports a role of RBP4 in transferring retinol from the parenchymal cell to the HSC (see papers by Senoo H, and others) but this is not what the introduction says. This statement and its supporting references need to be corrected.

The reviewer's comment is correct. We corrected the sentence accordingly (page 3 in the revised manuscript); 'Vitamin A (retinol), acquired from the diet, is transported to the liver and taken up by hepatocytes as a chylomicron remnant. It has been suggested that retinol binding protein (RBP) plays a role in the transfer of retinol from hepatocytes to HSCs via a RBP receptor STRA6.'

The statement on pages 3-4 including "effects of retinoids, including all-trans- and 9-cis- and 13-cis retinoic acid, are primarily mediated by two families of nuclear receptors, retinoic acid receptor (RAR) and retinoid X receptor (RXR) (Balmer & Blomhoff, 2002)" is partially incorrect. The action of 13-cis RA has never been shown to be mediated by RARs, and 9-cis-RA is now dubious as a physiological ligand of the RXRs. The statement and references need to be corrected and updated.

The reviewer's comment is correct, and we corrected the sentence accordingly (page 4); 'The diverse effects of retinoids are primarily mediated by two families of nuclear receptors, retinoic acid receptor (RAR) and retinoid X receptor (RXR) (Huang et al, 2014).'

Results, Page 4. "The effects of exogenous retinoic acid (RA) on HSCs or liver fibrosis are controversial; several studies showed that RA inactivated HSCs and alleviated hepatic fibrosis, while other reports showed the opposite (Friedman, 2008; Zhou et al, 2012)." --Please place the references immediately after the positions they support so that it is clear which shows which effect. If these are reviews, please use primary references to support. (also on page 10.)

We changed the references accordingly in page 4, 10.

"HSCs after passage 1 (HSCs-P1; activated HSCs) were incubated with citral, an inhibitor of retinaldehyde dehydrogenases (RALDH) that irreversibly convert retinaldehyde to RA, and examined for changes. Citral treatment increased cytoplasmic lipid droplets (Fig. 1A), as assessed by oil red O staining, and decreased a-SMA levels (Fig. 1B)..," --The cells should be extracted and tested for their content of retinyl esters. Citral, as a lipid aldehyde, is not just specific for inhibition of RALDHs, and it could be that the droplets are lipids and therefore they stain with Oil Red O, but they might not necessarily be composed of retinyl esters. This should be confirmed biochemically. The methods and legend also need to clarify how much retinol was present in the medium as a source of retinyl ester formation. If there are really increased retinyl esters in the HSC cells then there should be a commensurate reduction in retinol to account for this.

We examined whether retinyl palmitate levels are changed in citral-treated cells by reverse-phase HPLC [J Lipid Res. 2000 Jun;41(6):882-93] but found no significant increase. The reason may be that the detection method is not sensitive enough to detect small change. In this experiment, retinol and palmitate were not added to the culture media. Alternatively, the reappearance of lipid droplets may not accompany new synthesis of retinyl esters. At present, we have no clear answer for this. Thus, we tested whether reappeared lipid droplets exhibit autofluorescence as retinoid-containing lipid droplets exhibit a rapidly fading blue-green autofluorescence when excited with light of approximately 330 nm. There was a significant increase in autofluorescence in citral-treated cells, indicating that lipid droplets likely contain retinoids. This is included in the revised manuscript (page 12 and Fig. 1A). Regarding the retinol levels in the culture medium, we were also unable to detect significant changes. Although we did not reveal the source of retinyl esters in reappeared lipid droplets, that is, whether they were derived from retinol esterification or preexisting retinyl esters in cytoplasm, we have addressed the main issue that reappeared lipid droplets contain retinoids.

Page 5, "Intriguingly, siRNA treatment (RALDH1 and -2) also increased lipid droplets (Fig. S2) and decreased a-SMA levels and cell proliferation rate (Fig. 1E and S3)." -- Again, the implication is that this is due to differences in retinoids but the investigators need to assure their readers that it is not due to differences in other lipids. The statement that follows which says this "agrees with the recent paper that suppression of alcohol dehydrogenase (ADH) 3 inhibits HSC activation (Yi et al, 2014)." actually would support any lipid alcohol as being involved, as these classes of enzymes generally metabolize more than one substrate. The use of AGN193109 as a retinoid signaling antagonist is reassuring but there is no experiment showing that all-trans-RA can "rescue" the activated phenotype. This should be added.

As stated in response to the citral-treated cells above, we found no significant differences in retinyl palmitate levels but observed an increase in autofluorescence in siRNA-treated cells (Fig. S2), indicating that reappeared lipid droplets likely contain retinoids. We also examined whether all-trans RA could rescue the activated phenotype. Co-treatment of HSCs with all-trans-RA indeed reversed the effects of RAR antagonist, which is included in the revised manuscript (page 6, Fig. 1G and 1H).

Page 6, "Luciferase assay revealed that albumin expression decreases RA signaling by 70% (Fig. 2A)." --This is interesting but the statement should include the concentration of RA that was added and, if possible, the molar ratio of albumin to RA in the cells. It is not possible to infer whether this is due to the binding and therefore the effective removal of RA by albumin or to another effect.

To this end, we added different amount of R-III to HSCs and measured the effect on RA signaling by luciferase assay. R-III was found to decrease RA signaling in a dose-dependent manner, indicating that R-III effect is due to the removal of RA. This is included in the revised manuscript (Fig. 2A, right panel). When we performed luciferase experiment (Fig. 2A, left panel), RA was not added to culture medium.

Fig 2B: 5µM RA was used. Were lower concentrations also tested? 5 µM is far above the plasma level of, e.g, 10-50 nM. The relevance of this in vitro experiment would be greater if a more physiological concentration was tested and shown to be effective.

We have previously carried out this experiment with different concentrations of RA (50nM, 0.5 or 5mM) and got similar results. We changed the molar concentration to 50nM (page 22).

"...while co-expression of RALDH1 and -2 abolished the albumin effect, in addition to increasing cellular RA levels (Fig. 2D)." As above, as the RALDHs need substrate to produce RA, the text should specify how much retinol was in the medium and how much retinyl ester the HSCs-P1 contained. As the activation process results in the loss of retinyl esters, the longer-cultured primary HSCs might not be able to produce RA efficiently. Have the authors conducted similar experiments with activated HSCs?

Again, as stated in response to the citral-treated cells above, no significant changes in retinyl palmitate levels were observed in transfected cells. There was, however, an increase in autofluorescence in albumin-transfected cells as previously reported [Gut. 2009 Oct;58(10):1382-90], indicating that reappeared lipid droplets in albumin-transfected cells contain retinoids. We have transfected HSCs after passage 1, 2 and 3 with albumin expression vector and found that the phenotypic change in longer-cultured HSCs was not as dramatic as that seen in HSCs-P1.

"This suggests that the anti-fibrotic effect of albumin and R-III is largely mediated by downregulation of RA signaling." Since the HSC-P1 cell stage is most likely prefibrotic, it may be an earlier event that is being regulated.

We agree with the reviewer's concern and have rewritten the sentences carefully not to limit the potential actions of albumin/R-III to the downregulation of RA signaling throughout the manuscript.

"Lastly, previous reports showed that albumin readily binds to retinoic acid, possibly at fatty acid binding sites (Belatik et al, 2012)." The binding of RA to albumin was shown decades ago and that reference should be added (Smith et al, J Biochem 1973).

We added the reference as pointed out by the reviewer (page 6).

"Cellular uptake of R-III into HSCs is STRA6-dependent" (Fig. 3) - -This section focuses on STRA6, which, as the authors have noted, is not "robustly" expressed in the liver. But the RBP4-R2 receptor described by Alapatt et al (J Biol Chem 2013) might be a better candidate. The RNA collected from these studies should be examined for the expression of this alternative receptor for RBP4. The discussion actually states that the uptake of R-III was "mediated" by STRA6. This was never shown and the statement is too strong. A better model would be to use cells from liver of STRA6 knock-out mice. The authors should add experiments or modify their statement.

We agree with the reviewer that the role of RBPR2 also needs be examined in HSCs. However, in order to measure RBPR2 mRNA levels in HSCs or test whether siRNA-RBPR2 blocks R-III uptake, rat RBPR2 mRNA sequence is necessary but currently not available. Thus, we rewrote the sentences carefully, as suggested by the reviewer.

1. the subtitle in page 7 'Cellular uptake of R-III into HSCs is STRA6-dependent' → 'STRA6 may be involved in the cellular uptake of R-III into HSCs'.

2. the sentence in page 7 'To assess the mechanism of STRA6-mediated R-III uptake,..' → 'To test the possibility that STRA6 may be involved in R-III uptake,..'

3. the sentence in the discussion (page 10) 'Our in vitro experiments in this study showed that R-III uptake into HSCs was mediated primarily by STRA6, a RBP receptor, indicating that ...' → 'Our in vitro experiments in this study showed that R-III uptake into HSCs was markedly affected by siRNA-STRA6, indicating that …

Page 8, The studies using R-III in vivo are quite interesting and the difference in the appearance of the livers is quite striking. Do the investigators know the half-life of R-III in mouse plasma?

We measured the plasma half-life of R-III and its $T_{1/2}$ (~20 h) was included in the revised manuscript (page 13 and Fig. S8).

"In normal livers, desmin-positive, quiescent HSCs were distributed in the hepatic parenchyma within the perisinusoidal space, while desmin/a-SMA-positive, activated HSCs were primarily located in septal and portal areas in fibrotic livers (Fig. 6)." -- In Fig. 6, the distributions of desmin and a-SMA actually look rather different. Do they both represent HSCs? Why isn't the overlap closer?

We agree with the reviewer's opinion. As the liver sections appear to be overstained, possibly due to use of too high concentration of anti-desmin antibody, we repeated IHC staining. New desmin staining clearly shows HSCs and exhibit better overlap with that of a-SMA (Fig. 7).

It is stated that paired statistical analysis was performed. What were the pairs? It looks like the mouse study must have used groups of mice, not pairs. For some data, such as shown in Fig. 1D, 3B, S3, no statistics are shown. Please include.

The reviewer is correct about the paired statistical analysis. We rewrote the sentence in page 17 (in materials and methods); 'Paired t-test or two-sample t-test were preformed where appropriate'. The revised figures 1D, 3B, and S3 have statistics.

Other comments—
Information that is necessary in order for the study to be reproduced by others: Sex of mice; type and source of the diet fed; method and agent of euthanasia; length of culture before each passaging of HSC; composition of the HSC culture medium.

We have added these information in the revised manuscript.

Page 12: It is stated that "the purity of HSC was assessed by lipid droplets...", which to some extent wouldn't they also be present in parenchymal cells? What controls were used to rule out or quantify parenchymal cell contamination of the HSC?

The lipid droplets in hepatocytes can be considered negligible in size compared to those in hepatic stellate cells. As described in the previous manuscript, in order to assess the contamination of hepatocytes in the preparation of stellate cells, we performed Western blot analysis using antibody against hepatocyte-specific marker tyrosine aminotransferase (TAT) (page 5, Fig. S1). We have added this also in the Materials and Methods section of the revised manuscript (page 13).

What STRA6 antibody was it? The specificity of anti-STRA6 #26-445 from ProSci is listed as H (human), not rat or mouse. Was this the antibody that was used? Likewise, the catalogue numbers of the other antibodies should be listed. Negative staining controls should also be described.

We have used anti-STRA6 antibody (Prosci #46-715), which has species reactivity against rat, mouse STRA6. The catalogue numbers for other antibodies were included in the revised manuscript (page 13). Regarding the specificity of anti-STRA6 antibody, we used NIH3T3 cell lysates as a negative control, which is included in the revised manuscript (Fig. 3A).

"intravenously administered with saline, albumin, RBP or His-tagged R-III once per day during the last 2 weeks of CCl4 treatment." (also page 14). Please specify the site of i.v. injection and whether anesthesia was used. If so, was there a separate anesthesia control group or were the control mice injected with a placebo by the same route?

As suggested by the reviewer, we specified the injection site 'tail vein injection' in page 8 and 15. Anesthesia was not used for injection (page 15), and the control mice were given saline solution by the same route as written in the manuscript.

Minor points needing correction: please search for and correct--
Real-time RCR analysis, Grammar, ATRA

We have corrected mistakes that the reviewer pointed out.

Referee #2

CCl_4 represents a model of toxic liver injury. Other, more inflammatory model should also be tested.

We agree with the reviewer's opinion and have examined the therapeutic potential of R-III using bile duct ligation model of hepatic fibrosis. Administration of R-III attenuated cholestatic liver fibrosis and reduced collagen content by up to ~50%. Results from animal study are included in the revised manuscript (page 9 and Fig. 9).

1) Is the effect of R-III dose dependent?

We tested dose effects of R-III *in vitro* as well as *in vivo*. First, different amount of R-III was added to HSCs after passage 1 and its effect on RA signaling was measured by luciferase assay. R-III decreased RA signaling in a dose-dependent manner, which is included in the revised manuscript (Fig. 2A). In the animal study using bile duct ligation model, we also found that R-III (1, 5 or 10mg) exerted dose-dependent therapeutic effect against liver fibrosis (Fig. 9).

2) The authors show an enrichment of R-III in HSC within the liver, is R-III also observed in other organs?

We have previously reported that R-III, when intravenously injected, is detected also in extrahepatic organs such as brain, lung, spleen, pancreas, kidney and intestine (Choi et al, 2012). This is included in discussion section (page 12).

3) Are there any side effects of R-III treatment?

Mice that received R-III (10mg, intravenous injection once daily for 7 days) showed no apparent side effects. This is included in the discussion section of the revised manuscript (page 13).

4) Does the inhibition of stellate cell activation also inhibit inflammatory processes in liver fibrosis e.g. infiltration of the liver by immune cells?

To this end, we have stained liver sections for macrophage using anti-F4/80 antibody. F4/80 staining was increased in CCl_4-induced fibrotic liver, whereas R-III treatment reduced infiltration of macrophages. This result is included in the revised manuscript (page 8 and Fig. 6).

5) Is there any effect on classical fibrotic pathways such as the TGF-beta/ SMAD pathway?

We have stained liver section using anti-TGF-b antibody. TGF-b staining was markedly increased in CCl_4-induced fibrotic liver, while R-III treatment reduced its staining (page 8 and Fig. 6).

6) Is the effect specific for toxic models of liver fibrosis, which features only some of the complex aspects driving liver fibrosis in the human, thus can the results on liver fibrosis be reproduced e.g. in metabolic and more inflammatory models of liver fibrosis?

As described above, we observed that R-III also reduced bile duct ligation-induced liver fibrosis (Fig. 9).

2nd Editorial Decision 17 February 2015

Thank you for the submission of your revised manuscript to EMBO Molecular Medicine. We have now received the enclosed reports from the referees that were asked to re-assess it. As you will see the reviewers are now globally supportive and I am pleased to inform you that we will be able to accept your manuscript pending the following final amendments:

1) Please abide by Reviewer 1's request to correct Figure 1B and the mouse gene nomenclature

***** Reviewer's comments *****

Referee #1 (Remarks):

In this revised version, the authors have made a number of changes that, together, have addressed previous concerns and strengthened the manuscript.

1. The introduction now includes appropriately corrected background knowledge on vitamin A uptake into the liver. Information on RAR/RXRs is also corrected. (It might be noted however that the citation to Huang et al, 2014, is a recent review but these concepts have been known much longer and previous reviews could have been cited.)

2. The major revisions are the inclusion of a second model of liver fibrosis. In the previous review, other models were suggested (such as a viral model) but no specific model was requested. The authors chose to use a model of bile duct ligation-induced liver fibrosis (data in new Fig. 9). This model has some relevance to liver disease in humans and thus is considered an acceptable choice. The R-III fusion protein also reduced liver fibrosis in this model. The change does not appear to be as dramatic as with CCl_4-induced fibrosis, and it may be that this model is relatively "mild." However, having the two different studies, one with CCL4 and the new one with bile duct ligation, does help to strengthen the case that R-III could be a promising therapy at least in a mouse model.

3. The investigators also included new in vitro studies on the role of STRA6 in the uptake of the R-III protein into cultured hepatic stellate cells (HSC). They showed reduced uptake when siRNA for STRA6 was transfected (western blotting, Fig 3D). This experiment is quite interesting because the role of STRA6 has been considered a little controversial. (Debate on whether it is essential or not in mice did not address whether STRA6 actually performs a beneficial function in physiologically relevant stress situations.) Thus, this experiment adds to the understanding of how R-III may be taken up by HSC.

4. The investigators also examined the lipid droplets (stained with Oil Red O) for vitamin A content. They did not detect retinol or retinyl esters and suggest this may be because they did not add either as substrates to their cultures. The explanation is reasonable; however, the results also suggest that the lipid accumulation observed may not require or be directly related to vitamin A metabolism. While the interpretation of these new results is still up in the air, as the authors state in the discussion section, the inclusion of this analysis still helps to strengthen the manuscript.

5. Other comments in the previous review were the need for more detail on methods, so that the work can be reproduced by others. This has been addressed, for example in the legend to Suppl Fig 8.

In all, the authors have made a good-faith effort to improve the manuscript through new studies and presentation of additional data and via corrections and clarifications in the text.

(There is still a minor error in figure 1B where citrol should be citral. For mice, the appropriate gene nomenclature would be Aldh1a(1,2,3) rather than Raldh(1,2,3).

Referee #2 (Remarks):

The authors have responded to all my questions.

2nd Revision - authors' response 03 March 2015

Response to the requested amendments

1) Please abide by Reviewer 1's request to correct Figure 1B and the mouse gene nomenclature

We have corrected Figure 1B and replaced Raldh1/2 to Aldh1a1/2 in the revised manuscript and figures.
