# Supplementary figures and images for: Fusion protein of retinol-binding protein and albumin domain III reduces liver fibrosis
Source: EMBO Mol Med. 2015 Apr 11;7(6):819–30. doi: 10.15252/emmm.201404527 (PMC4459820; doi:10.15252/emmm.201404527)

Figure 2E:  $\alpha$ -SMA

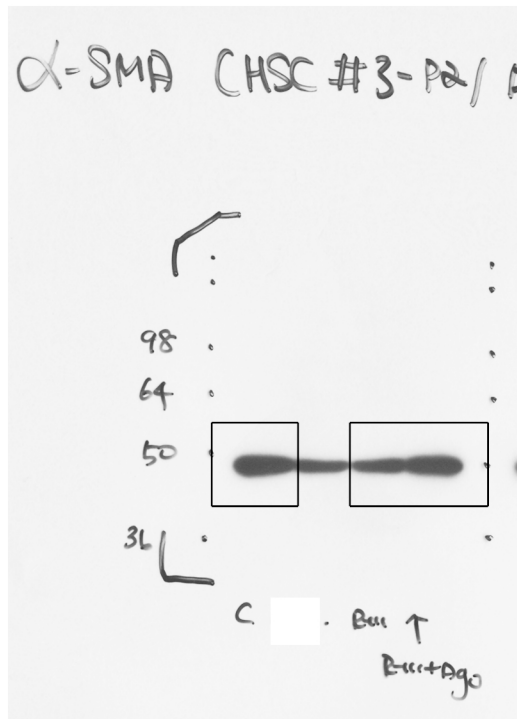

Figure 2E: His-tag

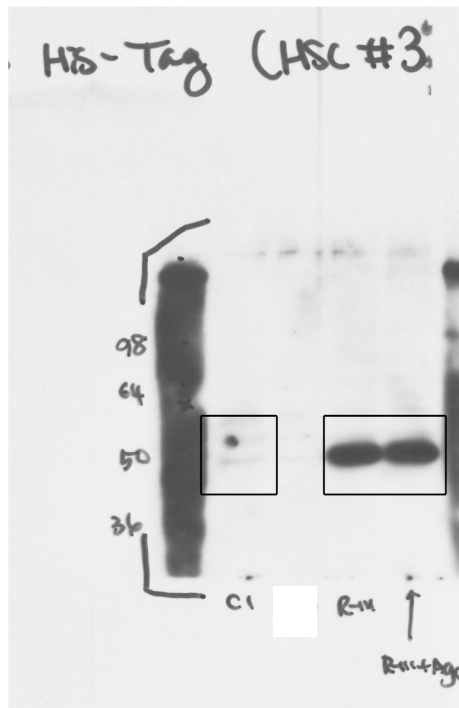

Figure 2E: tubulin

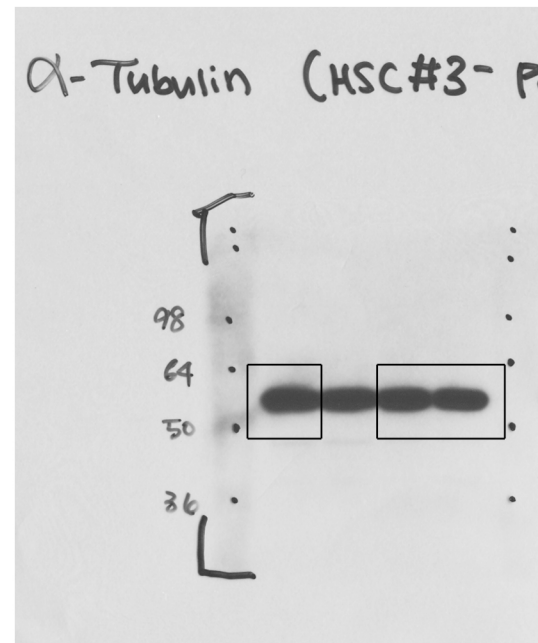

Supplement: Supplementary file 3 [file emmm0007-0819-sd3.pdf]

Fig. 3A STRA6

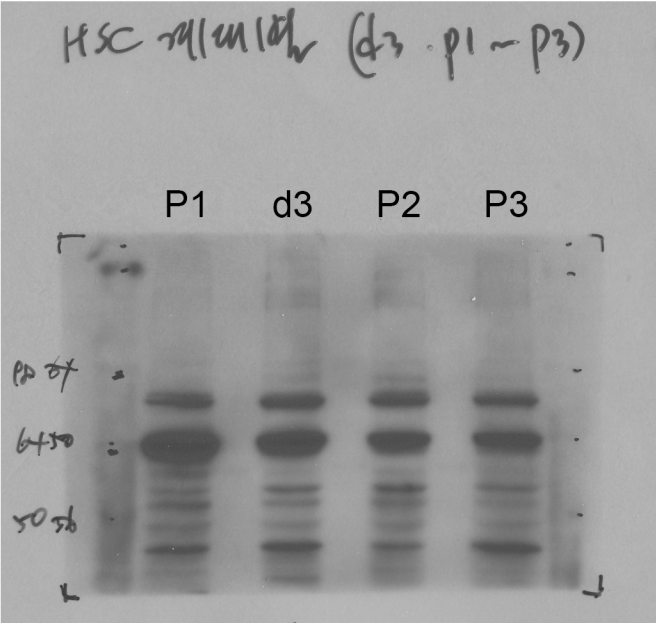

Fig. 3A tubulin

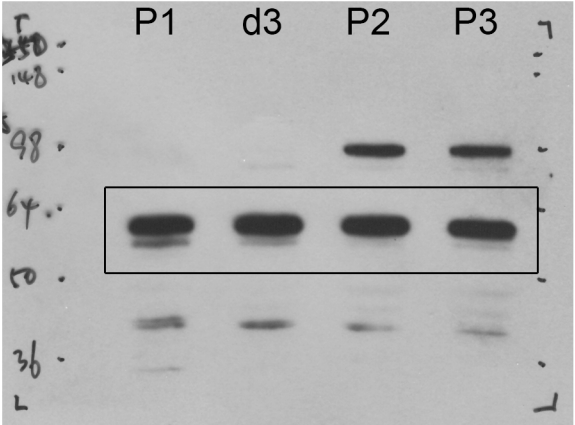

Fig. 3C his-tag

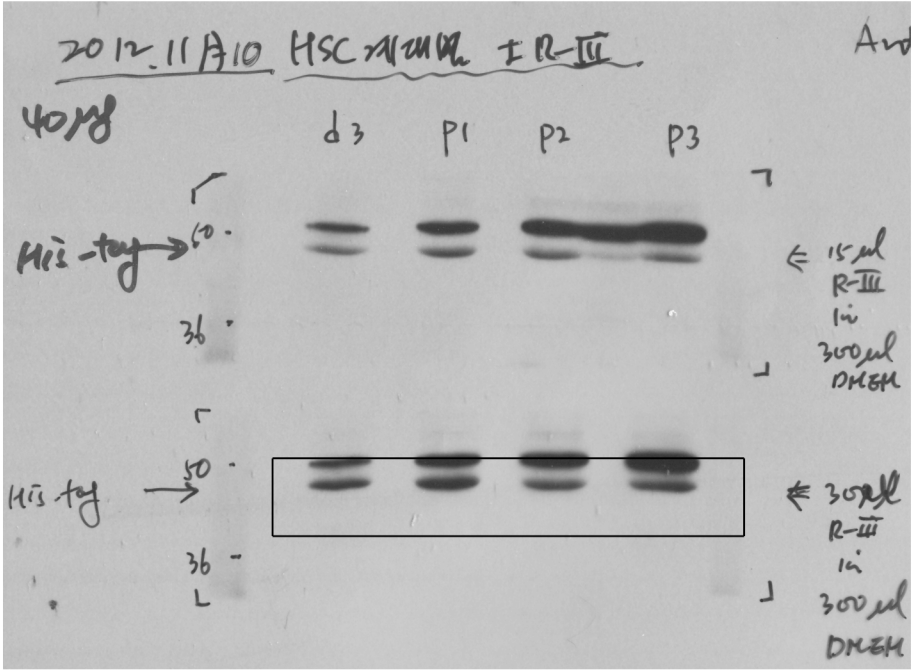

Fig. 3D his-tag

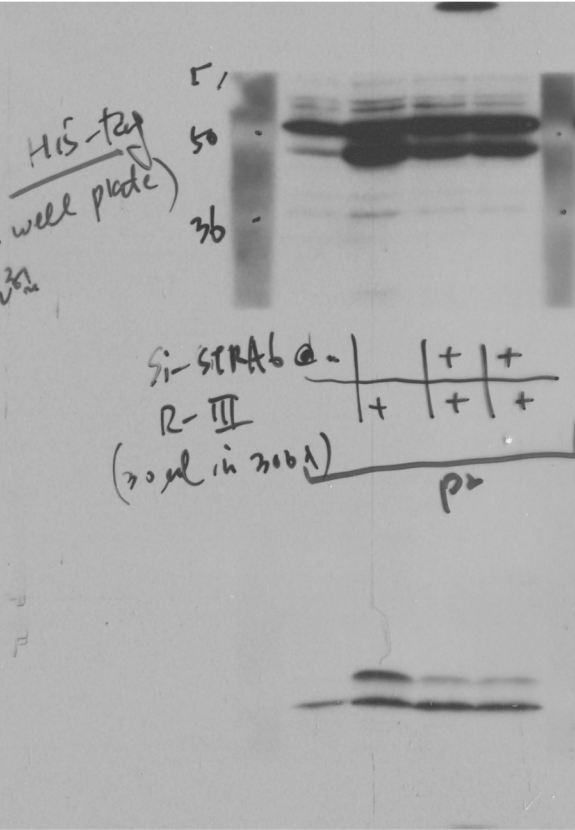

Fig. 3C tubulin

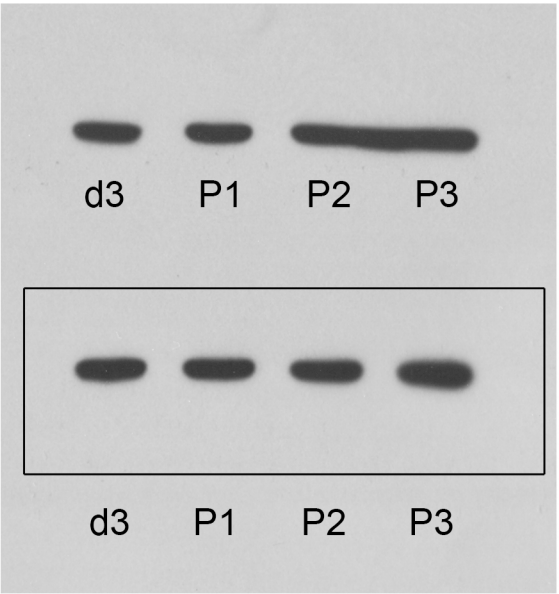

Fig. 3D STRA6

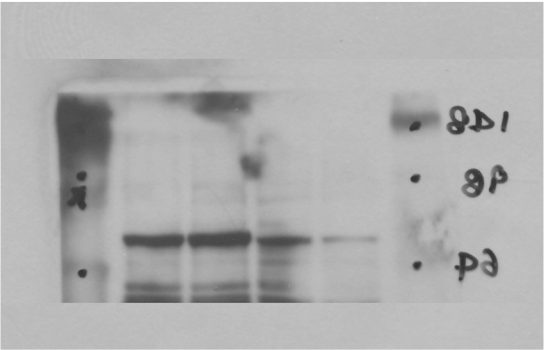

Fig. 3D tubulin

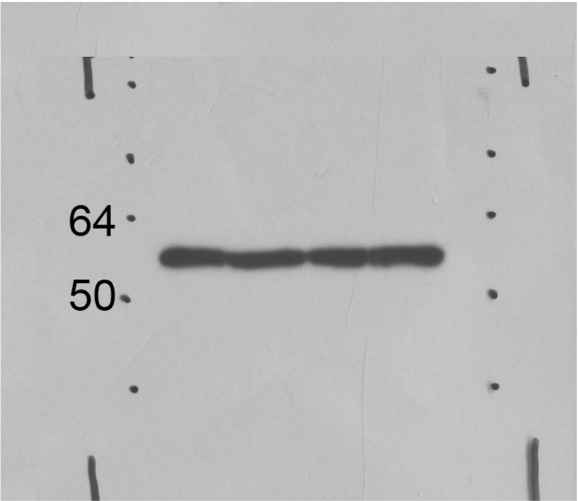

Supplement: Supplementary file 4 [file emmm0007-0819-sd4.pdf]
